# SeedTransNet: a directional translational network revealing regulatory patterns during seed maturation and germination

**DOI:** 10.1093/jxb/erac394

**Published:** 2022-10-08

**Authors:** Bing Bai, Bastian Schiffthaler, Sjors van der Horst, Leo Willems, Alexander Vergara, Jacob Karlström, Niklas Mähler, Nicolas Delhomme, Leónie Bentsink, Johannes Hanson

**Affiliations:** Umeå Plant Science Center, Department of Plant Physiology, Umeå University, SE-901 87 Umeå, Sweden; Wageningen Seed Science Centre, Laboratory of Plant Physiology, Wageningen University, 6708 PB Wageningen, The Netherlands; Umeå Plant Science Centre, Department of Forest Genetics and Plant Physiology, Swedish University of Agricultural Sciences, Umeå, Sweden; Department of Molecular Plant Physiology, Utrecht University, 3584 CH Utrecht, The Netherlands; Wageningen Seed Science Centre, Laboratory of Plant Physiology, Wageningen University, 6708 PB Wageningen, The Netherlands; Umeå Plant Science Centre, Department of Forest Genetics and Plant Physiology, Swedish University of Agricultural Sciences, Umeå, Sweden; Umeå Plant Science Center, Department of Plant Physiology, Umeå University, SE-901 87 Umeå, Sweden; Umeå Plant Science Center, Department of Plant Physiology, Umeå University, SE-901 87 Umeå, Sweden; Umeå Plant Science Centre, Department of Forest Genetics and Plant Physiology, Swedish University of Agricultural Sciences, Umeå, Sweden; Wageningen Seed Science Centre, Laboratory of Plant Physiology, Wageningen University, 6708 PB Wageningen, The Netherlands; Umeå Plant Science Center, Department of Plant Physiology, Umeå University, SE-901 87 Umeå, Sweden; Instituto de Agrobiotecnología del Litoral, Argentina

**Keywords:** *Arabidopsis thaliana*, mRNA regulation, ribosome, seed germination, seed maturation, translatome profiling

## Abstract

Seed maturation is the developmental process that prepares the embryo for the desiccated waiting period before germination. It is associated with a series of physiological changes leading to the establishment of seed dormancy, seed longevity, and desiccation tolerance. We studied translational changes during seed maturation and observed a gradual reduction in global translation during seed maturation. Transcriptome and translatome profiling revealed specific reduction in the translation of thousands of genes. By including previously published data on germination and seedling establishment, a regulatory network based on polysome occupancy data was constructed: SeedTransNet. Network analysis predicted translational regulatory pathways involving hundreds of genes with distinct functions. The network identified specific transcript sequence features suggesting separate translational regulatory circuits. The network revealed several seed maturation-associated genes as central nodes, and this was confirmed by specific seed phenotypes of the respective mutants. One of the regulators identified, an AWPM19 family protein, PM19-Like1 (PM19L1), was shown to regulate seed dormancy and longevity. This putative RNA-binding protein also affects the translational regulation of its target mRNA, as identified by SeedTransNet. Our data show the usefulness of SeedTransNet in identifying regulatory pathways during seed phase transitions.

## Teaser

We describe the dramatic shifts in translation that occur during seed maturation, germination, and seedling establishment. Using network analysis, we identify putative regulatory relationships and subsequently confirm some of them *in vivo*.

## Introduction

In higher plants, seed development is generally divided into two phases, embryogenesis and seed maturation. In Arabidopsis, embryogenesis starts from double fertilization occurring in the ovule and ends when cell division arrests. This period is followed by a seed maturation phase during which metabolic reserves such as sugars, proteins, and lipids are deposited in the endosperm and embryo (Baud *et al.*, 2002, 2008). After this, seeds undergo desiccation, during which metabolism gradually ceases. The eventual quiescent state allows the seed to survive until it is able to germinate. A subset of mRNAs, produced during seed maturation, are stored in the form of messenger ribonucleoprotein (mRNP) complexes (Peumans *et al.*, 1979) These RNAs retain their functions during dry storage and are translated upon imbibition (Hughes and Galau, 1989; Comai and Harada, 1990; Beltrán-Peña *et al.*, 1995; Bai *et al.*, 2017).

Regulation of translation provides an important way of determining final cellular protein levels. The average ribosome occupancy of a transcript is a proxy for translational activity, independent of any local variation in translation speed (Li *et al.*, 2014). Moreover, an imbalanced production of the components of a protein complex will lead to its misfolding or degradation. Thus, protein complex components must be translated in a coordinated manner in order to efficiently allocate resources. Several studies on polysome occupancy of mRNAs in seeds have shown distinct translational regulatory activities (Layat *et al.*, 2014; Basbouss-Serhal *et al.*, 2015; Bai *et al.*, 2017). Analysis of the mRNAs that are under translational regulation has revealed several different sequence features that correlate with translational control, indicating that several translational regulatory mechanisms operate in parallel during seed germination (Bai *et al.*, 2017).

The seed maturation period is of vital importance for the development of viable seeds. When the seed is fully matured, it forms a resilient dry structure capable of carrying the plant through harsh conditions and time. The stability of the seed has allowed flowering plants to dominate the world’s land, and today this group is of vital importance for all terrestrial ecosystems as well as agriculture. From the perspective of the biological activity of the seed, the dry period represents absence of activity and it is therefore possible to envisage the trajectory from fertilization to seedling as being a single timeline with a quiescent interruption. The transcriptional events that take place during this timeline are well characterized (Le *et al.*, 2010; Dekkers *et al.*, 2013; Yi *et al.*, 2019). In Medicago, network analyses on global transcription interactions identified distinct co-expression modules related to the acquisition of desiccation tolerance, longevity, and pod abscission, indicating that distinct regulatory programmes are at play during seed maturation (Verdier *et al.*, 2013). Transcript level changes have also been studied thoroughly in Arabidopsis (Angelovici *et al.*, 2009; Le *et al.*, 2010; Belmonte *et al.*, 2013); however, little is known about how translation is regulated at later stages (Layat *et al.*, 2014; Basbouss-Serhal *et al.*, 2015; Bai *et al.*, 2017). In this study, we investigated translational dynamics during seed maturation and integrated our findings into network analysis with our previously published data from seed germination and seedling establishment (Bai *et al.*, 2017). We discovered that genes are extensively translationally regulated during early seed maturation whereas only transcript-level changes were detected during late maturation. Our combined network analysis (SeedTransNet) identified several regulatory modules with genes sharing both biological functions and regulatory elements. We were able to experimentally confirm the identified node genes as regulators of seed physiology by examining the seed germination phenotypes of their respective mutants. We also identified a new putative translational regulator, PM19L1, and confirmed its regulatory potential *in vivo*.

Viewing seed development, maturation, germination, and seedling establishment as a single temporal trajectory is thus a fruitful approach, and SeedTransNet can most probably be mined for regulators of several seed-specific processes.

## Materials and methods

### Plant material and growth conditions

Maturing *Arabidopsis thaliana* seeds (accession Columbia-0) were grown as three biological replicates of 50 plants each. Plants were grown on 4 × 4 cm Rockwool blocks in a growth chamber at 20 °C/18 °C (day/night) under a 16 h day/8 h night photoperiod of artificial light (150 μmol m^–2^ s^–1^) and 70% relative humidity. Plants were grown in a standard nutrient solution (He *et al.*, 2014). Flowers were tagged at each time point after flower opening and pollination. Seeds were harvested at each specified developmental stage including 12, 15, 18, and 20 days after flowering (DAF). Seeds of the *abi3-5* mutant were harvested at the time when the corresponding wild-type seeds were fully matured (Dekkers *et al.*, 2016.). About 200–400 mg of seed were harvested and frozen in liquid nitrogen, freeze-dried, and stored at –80 °C until required for further analysis. To collect maturing seeds, we tagged the plants at each developmental stage and harvested whole siliques into liquid nitrogen. After freeze-drying, we released the seeds from each silique with gentle pressure on the silique, and purified the seeds by filtering with sieves. Only the seed material was used for polysome profiling. All mutant seed used were obtained from Arabidopsis stock centres, checked for single site integration by segregation analysis, and selfed until homozygosity was attained before experiments were performed.

### Isolation of total and polysomal RNA, and polysome analysis

Isolation of total and polysomal RNA was performed according to Bai *et al.* (2017). Ribosomes were purified using ~400 mg of freeze-dried ground tissue extracted with 8 ml of polysome extraction buffer (PEB; 0.25 M sucrose, 400 mM Tris, pH 9.0, 200 mM KCl, 35 mM MgCl_2_, 5 mM EGTA, 50 μg ml^–1^ cycloheximide, 50 μg ml^–1^ chloramphenicol). Each extract (10 ml) was loaded on top of an 8 ml sucrose cushion (1.75 M sucrose in PEB) and centrifuged (18 h, 90 000 *g*) using a Beckman Ti70 rotor (Beckman Coulter, Brea, CA, USA). The resulting pellet was resuspended in 0.5 ml of suspension buffer (200 mM Tris, pH 9.0, 200 mM KCl, 0.025 M EGTA, 35 mM MgCl_2_, 5 mM DTT, 50 μg ml^–1^ cycloheximide, 50 μg ml^–1^ chloramphenicol), loaded on a 4.5 ml 20–60% linear sucrose gradient, and centrifuged at 190 000 *g* for 1.5 h, at 4 °C using a Beckman SW55 rotor (Beckman Coulter). After ultracentrifugation, each gradient was fractionated into 20 fractions using a Teledyne Isco Density Gradient Fractionation System (Teledyne Isco, Lincoln, NE, USA) with online spectrophotometric detection (254 nm). The fractions corresponding to the polysome region in the ribosome profile were pooled for further analysis. The ribosome abundance, which is reflected in the area under the curve, was calculated after subtracting the baseline obtained by measuring a blank gradient and normalizing to the total area under the curve to account for possible uneven loading of the gradients.

### Data analysis

Affymetrix Arabidopsis Gene 1.1 ST Arrays (Affymetrix, Santa Clara, CA, USA) were hybridized using the GeneChip® 3ʹ IVT Express kit (cat. # 901229) according to instructions from the manufacturer. Hybridization data were analysed and gene-specific signal intensities were computed using the R statistical programming environment (https://www.r-project.org), the BioConductor package affy (Gautier *et al.*, 2004), and the Brainarray cdf file ver. 17.1.0 (http://brainarray.mbni.med.umich.edu/). DNA microarray data are available in the Gene Expression Omnibus (GEO) repository (http://www.ncbi.nlm.nih.gov/geo/) under accession number GSE127509. The limma and affy packages were used for RMA normalization (Irizarry *et al.*, 2003). Probe sets with intensity signals that never exceeded the noise threshold (log_2_Exprs <4 in all samples) were removed. A linear Bayesian model was applied to assess differential expression using the limma package (Smyth, 2004). Polysome occupancy (PO) changes were considered significant if the overall PO changes exceeded a 2-fold change (FC) >1 and *P*-value <0.05 adjusted by the false discovery rate (FDR) according to the method of Benjamini and Hochberg (1995) (summarized in Supplementary Fig. S1). Principal component analysis (PCA) was performed using TM4 (Saeed *et al.*, 2003).

Gene set enrichment analyses were performed using an in-house utility: gopher; an algorithm developed at the Umeå Plant Science Center. For Gene Ontology (GO) enrichment, it uses the Parent–Child test (Grossmann *et al.*, 2007) and a Benjamini–Hochberg multiple testing correction (Benjamini and Hochberg, 1995). Significant terms were selected if their FDR was ≤0.05. Gene sets were compared with a background dataset consisting of sequences of all genes considered expressed at any developmental stage in the dataset.

### Sequence feature analysis

Genes with significantly increased or decreased PO at each translational shift were compared with all mRNAs expressed in the experiment as background for enrichment of sequence features using custom scripts. Different parts of the cDNAs [coding sequence (CDS), 5ʹ untranslated region (UTR), 3ʹUTR, and full transcript] were compared separately. The distributions of sequence length and GC content were evaluated separately for the CDS, 5ʹUTR, 3ʹUTR, and full transcript. CDSs were also analysed for GC3 content, measured using the effective number of codons (Nc) index (Sun *et al.*, 2013), after removing sequences lacking start codons and/or containing premature stop codons and CDSs shorter than 100 codons. The same analyses were performed separately for the CDSs of protein-coding genes having an annotated UTR and those lacking proper annotation (UTRs were called present when their length exceeded 1 nucleotide). Given the non-normality of the distributions of values, a Wilcoxon signed-rank test was adopted for all statistical comparisons (median as test statistic).

### RNA structure analysis

Experimentally determined structure scores per nucleotide, as provided by Li *et al.* (2012), were used to calculate average structure scores for the genes with significantly increased and decreased ribosomal association at each developmental switch. Relative scaling was achieved by averaging the structure scores per region (5ʹUTR, CDS, and 3ʹUTR) in 100 bins. Standard error calculations and Student’s *t*-tests were performed using the Python SciPy module (http://www.scipy.org/).

### Motif analysis

DNA motif analyses were performed using the MEME suite (Bailey *et al.*, 2009), for full transcript, 5ʹUTR, CDS, and 3ʹUTR sequences, extracted from the TAIR10 database (http://www.arabidopsis.org/). The minimum and maximum motif widths were set to six and 10, respectively. If a gene had multiple transcripts, only the TAIR10 representative splice form was used. Background dinucleotide frequencies were provided separately for each sequence type. To test the specificity of the resulting motifs, FIMO (Bailey *et al.*, 2009) was used to scan all genes represented on the microarray for motif hits in the corresponding sequence type. Motifs with a *P*-value <0.001 were considered to be significant hits. The motif counts obtained were used to compute the enrichment *P*-value for the gene lists versus all mRNAs expressed in the experiment as background by means of a one-tailed Fisher’s exact test, performed with a custom script and the R software package (http://www.r-project.org/). The occurrences were derived from the same FIMO outputs. For each motif, the positions on the transcripts, as provided by the FIMO output, were used to calculate the relative number of motifs per (relative) position along the mRNA. Relative scaling was performed in a similar fashion as for the structure scores.

### Data collection and network construction, inference, and visualization

In order to collect a dataset for a full assessment of translational dynamics during seed maturation and germination, DNA microarray data from Bai *et al.* (2017) were retrieved from the Gene Expression Omnibus (GEO) repository (http://www.ncbi.nlm.nih.gov/geo/) under accession number GSE65780 (http://www.ncbi.nlm.nih.gov/geo/query/acc.cgi?token=urelcaiqjnqbrux&acc=GSE65780), and GSE127509 (https://www.ncbi.nlm.nih.gov/geo/query/acc.cgi?acc=GSE127509) from the current study. The limma and affy packages were used for RMA normalization (Irizarry *et al.*, 2003) with R version 3.5.2. Only those genes that passed the noise filter (log_2_Exprs >4) in at least one developmental stage at either the transcriptional or the translational level were selected for further data processing. PO was calculated as the log_2_ ratio of polysomal RNA to total RNA according to Bai *et al.* (2017). PO profiles for three biological replicates were clustered using Euclidean distance and visualized with a heat map constructed in R using the package ‘pheatmap’ (https://CRAN.R-project.org/package=pheatmap). The number of clusters was determined by the R package ‘NbCluster’ with the ‘fviz_nbclust’ function (Charrad *et al.*, 2014).

The replicated PO dataset was used as input for network construction. For network inference, the Seidr toolkit was used for meta-network construction (Schiffthaler *et al.*, 2019, Preprint). In brief, nine gene network inference methods were run: ANOVA (Küffner *et al.*, 2012), Aracne2, CLR (Faith *et al.*, 2007), GeneNet (Opgen-Rhein and Strimmer, 2007), GENIE3 (Huynh-Thu *et al.*, 2010), NARROMI (Zhang *et al.*, 2013), Pearson, Spearman, and a modified implementation of TIGRESS (Haury *et al.*, 2012), and their results were aggregated into a consensus network using the Top1 method (Hase *et al.*, 2013). An assessment of the scale-free property of the consensus network, fitting a heavy-tailed distribution using a log–log linear model, was performed at several candidate thresholds in the range 0.9999 to 1 with a step size of 1e-5. The network transitivity [or average cluster criterion (ACC)] was calculated within the same range. The best threshold was chosen at 0.99997 with an *R*^2^ scale-free fit of 0.95 and an Akiake information criterion (ACC) of 0.4. The filtered network was visualized and processed using Gephi (https://gephi.org/). The network was further partitioned using Infomap (Rosvall and Bergström, 2008). The code for running Seidr and analysing its results is available from https://dx.doi.org/10.5281/zenodo.5659552.

### Seed phenotyping

Seeds were harvested in bulk from four plants for each biological replicate, four biological replicates for each genotype. Germination experiments were performed as described previously (Joosen *et al.*, 2010). In brief, two layers of blue germination paper were equilibrated with 50 ml of demineralized water in plastic trays (15 × 21 cm). Six samples of ~50–150 seeds were spread on wetted papers using a plastic mask to ensure accurate spacing. Stacked trays were wrapped in a closed transparent plastic bag. The experiment was carried out in a 22 °C incubator under continuous light (143 μmol m^−2^ s^−1^). Pictures were taken twice a day for a period of 6 d using the same camera and Germinator package software according to Joosen *et al.* (2010) to score the number of seeds germinating. To quantify seed dormancy (DSDS50: days of seed dry storage required to reach 50% germination), germination tests were performed weekly until all seed batches had germinated to at least 90%. A generalized linear model with a logit link as described by Hurtado *et al.* (2011) was adapted to calculate DSDS50. Germination data were adjusted by choosing *n*=100 and fitted as one smooth curve per line. The observed germination proportion was re-interpreted as having observed *y* ‘successes’ in *n* binomial trials (e.g. 75% germinated means *y*=75 out of 100 possible ‘trials’). DSDS50 is the closest time point to that where a horizontal line at *y*=50 crosses the fitted curve. To measure seed longevity, an artificial ageing test was performed by incubating seeds above a saturated ZnSO_4_ solution (40 °C, 85% relative humidity) in a closed tank with circulation for 5–9 d (according to the International Rules for Seed Testing, https://www.seedtest.org). The seeds were then taken out and germinated as described before.

## Results

### Ribosome profiling indicates changes in global translation during seed maturation

**Fig. 1.**
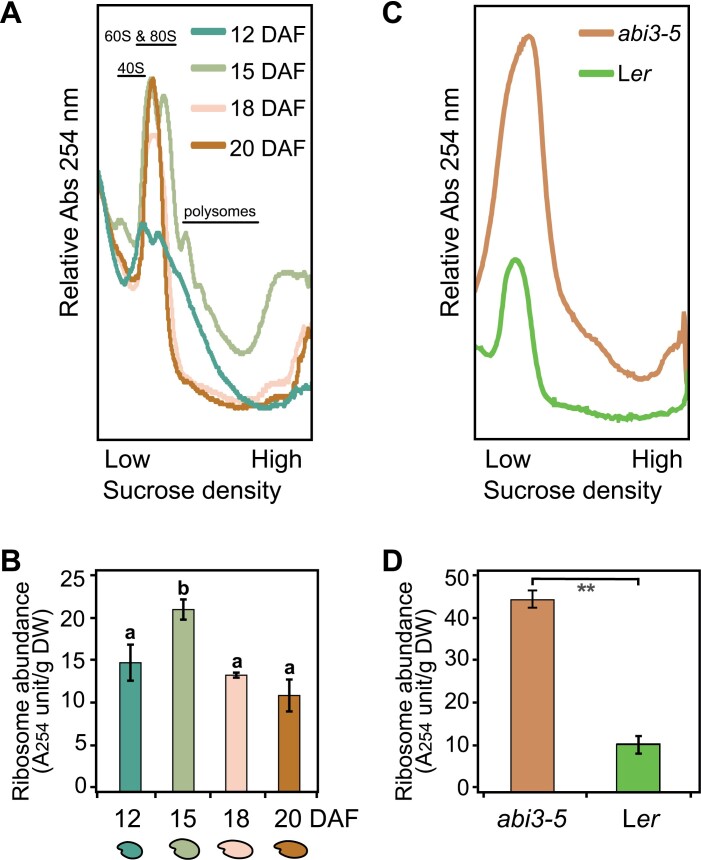
Ribosome and polysome abundance change during seed maturation. (A) Ribosome profiles from four developmental stages during seed maturation, 12, 15, 18, and 20 DAF (days after flowering). The profile is based on ribosome loading from equal seed dry weights. Approximate positions of ribosomal complexes are indicated. (B) Relative ribosome abundance at four developmental stages during seed maturation as determined by area under the curve in (A). Bars represent averages (*n*=3), error bars indicate ±SD, and the letters above each bar indicate the significance (Student’s *t*-test, *P*-value <0.05). (C) Comparison of ribosome profile of *abi3-5* mutant and wild-type seeds. Both seed types were harvested at the same time when wild-type seeds were fully matured. The profile is based on ribosome loading from equal seed dry weights. (D) Relative ribosome content of wild-type and *abi3-5* seeds as determined by the total area under the curve in the polysome and monosome regions of the density gradient in (C). Bars represent averages (*n*=3), error bars indicate ±SD, and asterisks indicate the significance (Student’s *t*-test, *P*-value <0.01).

To follow global translational activity during seed maturation, ribosomal profiling was performed on maturing Arabidopsis seeds at specific developmental stages: 12, 15, 18, and 20 DAF. At 12 DAF, seeds have just entered the maturation stage, and cell division has ceased; however, cells still expand, cotyledons and axis develop, and reserve accumulation is ongoing (Goldberg *et al.*, 1994; Le *et al.*, 2010). At this stage, ribosomes were present mainly in polysome form and only small peaks in the monosome region were visible (Fig. 1A). At 15 DAF, the embryo has reached its final volume, but reserve accumulation is still ongoing. The total amount of ribosomes, especially that of large polysomes, increased, indicating a global increase in translation (Fig. 1A, B). At 18 DAF, the seeds start to lose water to reach the desiccated stage. The monosome peak dominated, while the absorbance in the polysome region of the sucrose gradient was close to background level. No major changes could be seen between 18 and 20 DAF. During maturation, the overall ribosome abundance increased up to 15 DAF and thereafter decreased (Fig. 1B). To determine whether the accumulation of monosomes during seed maturation is developmentally regulated, we analysed the ribosomal profile of the seed maturation mutant *abscisic acid insensitive 3-5* (*abi3-5*), which displays a green seed phenotype. In the *abi3-5* mutant, the embryo fully develops whereas final maturation is impaired (Ooms *et al.*, 1993; Sugliani *et al.*, 2009). In the *abi3-5* seeds, a significantly higher ribosome abundance was detected compared with that in the wild-type Landsberg *erecta* (L*er*) (Fig. 1C, D), thus suggesting that a decrease in ribosomal abundance is regulated during seed maturation (Fig. 1A).

### Transcript level and translational changes during seed maturation

To investigate translational regulation during seed maturation, total (T) and polysomal-associated (P) mRNA was isolated from the four maturation stages and hybridized to microarrays. In total, 20 428 genes were expressed in any of the developmental stages (Supplementary Dataset S1). Thousands of genes changed transcript level during early seed maturation (12–15 DAF), whereas only a few hundred altered from 15 to 18 DAF. At the end of seed maturation (18–20 DAF), no significant transcript level changes were identified. About a third, 29% (3229 genes), of the genes shown to be changed at the transcript level were also identified as differentially expressed during maturation in another study (Angelovici *et al.*, 2009), partly validating our results (Supplementary Fig. S2A). Transcript level changes during maturation are conserved, since similar transcriptional changes were detected during *Medicago truncatula* seed maturation (Verdier *et al.*, 2013) (Supplementary Fig. S2B).

**Fig. 2.**
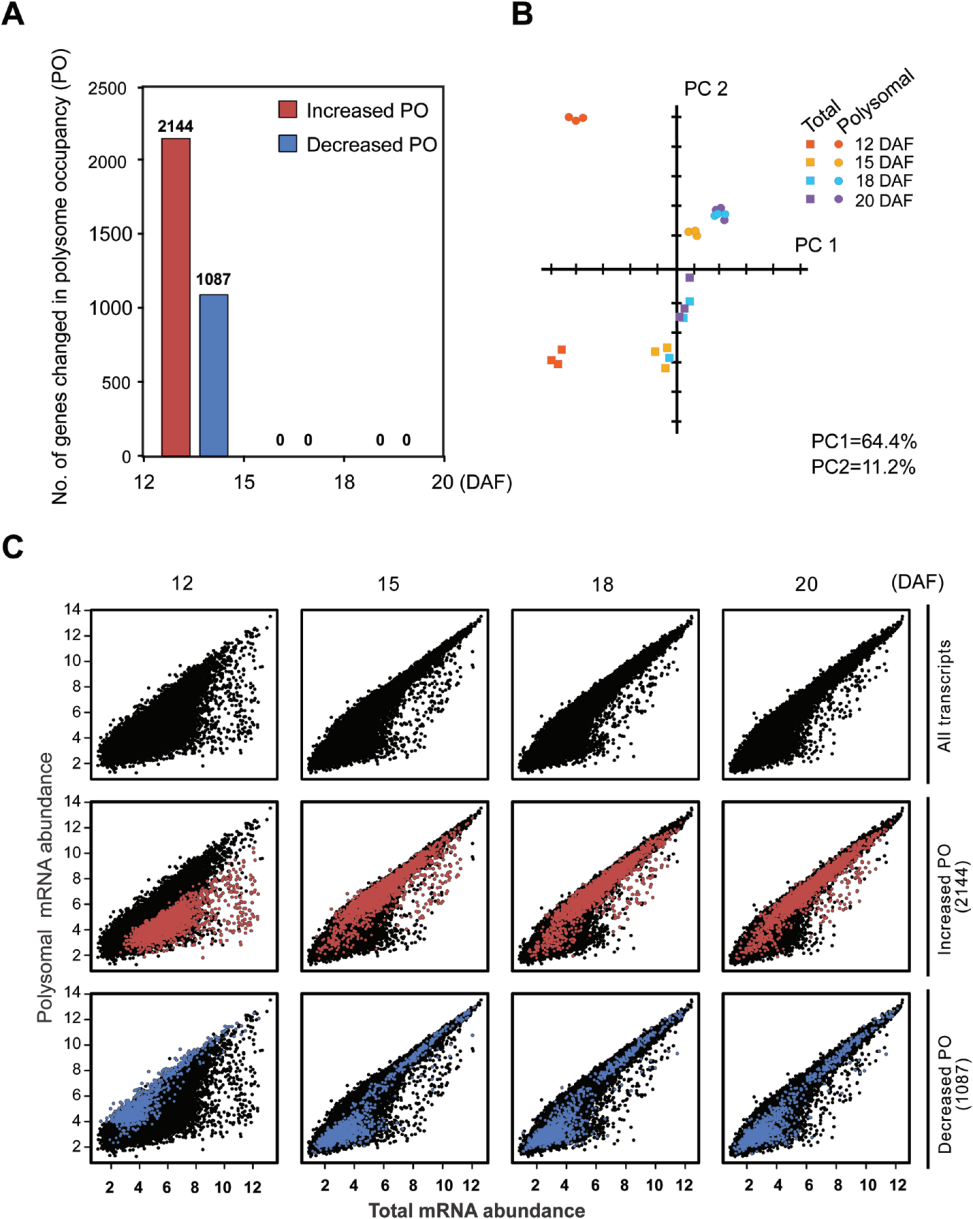
Translational shift during Arabidopsis seed maturation. (A) The number of genes showing changed polysome occupancy (PO) during seed maturation. Seeds were harvested 12, 15, 18, and 20 days after flowering (DAF). The increase or decrease in PO is indicated by red and blue bars, respectively, with gene numbers above the bars. Genes were scored as changing if the PO change compared with the preceding time point was >2-fold and associated with a corrected *P*-value of <0.05. (B) PCA of normalized expression levels for the total RNA (T) and polysomal RNA (P) during seed maturation. Individual biological replicates are highlighted in the same colour. The total RNA and polysomal RNA samples are indicated as circles and squares, respectively. The different colours represent different time points during seed maturation. The first two components, PC1 and PC2, explain, respectively, 64.4% and 11.2% of the total variation. (C) Relationship between normalized abundance of genes in the polysomal and total RNA preparations. Genes associated with significantly changed PO between 12 and 15 DAF (MTS genes) are indicated in red (increased PO) or blue (decreased PO).

To identify genes translationally regulated during seed maturation, the change in PO, defined as the ratio between the polysomal mRNA and total mRNA intensities of each stage, was determined. Significant PO changes for >3000 genes (log_2_ FC >1, corrected *P*-value <0.05) were identified between 12 and 15 DAF with significantly more changes in the positive direction, Fig. 2A (2144 genes increased in PO in contrast to 1087 genes with decreased PO) while only a few changes were detected after this stage (Supplementary Dataset S2). By analogy with the situation during seed germination, where translational regulation is concentrated at specific time points (Bai *et al.*, 2017), we term this change the maturation translational shift (MTS). This concentration of translational regulation to one time point is supported by PCA, where the relationships between the polysome samples and the total RNA samples are different in 12 DAF samples compared with those at the other time points (Fig. 2B). The translationally up-regulated genes have on average low transcript levels and are similarly associated at lower than average levels with the polysomes at 12 DAF (Fig. 2C). At 15 DAF, these genes were associated with polysomes at higher than average levels, and this trend persisted until the later stages. Thus, the PO increase is not a consequence of decreased mRNA levels (Fig. 2C). The down-regulated genes were more associated with polysomes at 12 DAF and detached from polysomes at 15 DAF. However, as only relative expression levels can be determined using microarrays, it is possible that the increase in relative level is caused by decreased mRNA stability or a general decrease in transcription, and the reverse may be true for the down-regulated genes. Several of the translationally regulated genes identified here have been shown to be translationally regulated during seed germination or in response to hypoxia stress (Supplementary Fig. S3A, B). GO analysis for the translationally regulated genes revealed that diverse molecular functions and biological processes were under translational control during seed maturation (Supplementary Dataset S3). The translationally up-regulated genes were enriched for GO terms including flavonoid biosynthesis, vegetative to reproductive phase transition, chromatin assembly, ABA (abscisic acid) metabolic process and signaling. Translationally down-regulated genes were enriched for functions including glycolytic process, fatty acid biosynthesis, gluconeogenesis, photosynthesis, and stress response.

### Coordinated translational dynamics during seed maturation and germination

**Fig. 3.**
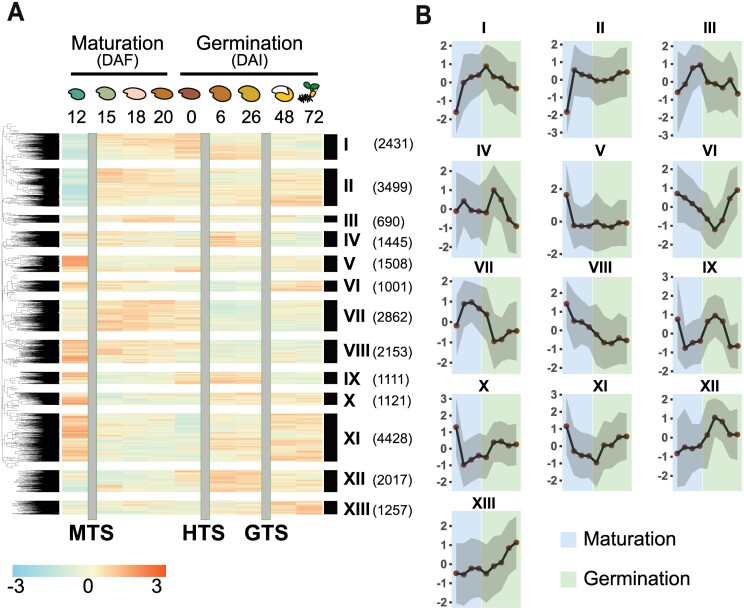
Dynamics of polysome occupancy (PO) during seed maturation and germination. The data used for the clustering analysis were combined from data presented in this study and the data from Bai *et al.* (2017). (A) Heatmap and hierarchical clustering of PO during seed maturation and germination for all genes present on the array. The time course included 12, 15, 18, and 20 days after flowering (DAF) from this study and after-ripened dry seeds (0) as well as seeds imbibed for 6, 26, 48, and 72 h (HAI) from the study by Bai *et al*, (2017)_._ The average PO of the three biological replicates is plotted. The colours represent relative PO on a log_2_ scale as indicated below the heat map. The 12 clusters are represented with Roman numerals. The number of genes in each cluster is indicated in parentheses. The grey bars that separate the developmental stages represent the three translational shifts [maturation translational shift (MTS), hydration translational shift (HTS), and germination translational shift (GTS)]. Heatmap colours indicate log_2_ fold change from –3 (blue) to 3 (red) as indicated below the heatmap. All genes considered expressed are included in the heatmap. (B) Trend plot of PO across the whole time course for each of the clusters identified in (A). The maturation and germination stages are indicated with a blue and a green background, respectively.

Previously, two distinct translational regulatory shifts (termed hydration translational shift, HTS, and germination translational shift, GTS, respectively) during seed germination have been identified (Bai *et al.*, 2017). In this study, we identified a single stage, at the early stage of seed maturation, where translation is regulated; the MTS. Plant development can be seen as one trajectory from embryogenesis to the mature plant; this development is interrupted by a quiescent period in the seed. Here, we treated the combined seed data, from early maturation to seedling development, as a single time course (Supplementary Dataset S4). Genome-wide PO profiles of the combined dataset, as proxy for gene translation efficiency throughout seed maturation and germination, displayed a dynamic pattern during seed maturation and germination (Fig. 3). The PO dynamics were represented by 13 clusters (I–XIII) in which the transcripts showed similar translational regulation (Fig. 3; Supplementary Dataset S5). Clusters I, III, VII, and XII represented transcripts with enhanced PO during seed maturation and reduced PO during seed germination; clusters V, VI, X, and XI contained transcripts with reduced PO during seed maturation and different dynamics during seed germination; clusters II and V contained transcripts with changes in PO only during seed maturation; and clusters VIII and XIII represented genes with PO that changed consistently across seed maturation and germination. As expected, transcripts from, for example, cluster III were enriched for transcripts classified under the biological processes ‘water deprivation’ and ‘embryo development ending in seed dormancy’, which were enhanced in PO during maturation and are important processes during seed maturation, while cluster IV and XI were enriched in transcripts involved in post-embryonic growth and seed germination, which increase in PO at a different stage during seed germination (Supplementary Dataset S6). The correlation between distinct regulatory patterns and discrete gene functions strengthens the biological relevance of translational regulation during the developmental transition studied. The large number of distinct expression patterns indicates that several distinct regulatory pathways are operational during the developmental trajectory. However, the co-expression clustering does not give any information about how these pathways are connected.

### Translational network analysis reveals regulatory patterns during seed maturation and germination

**Fig. 4.**
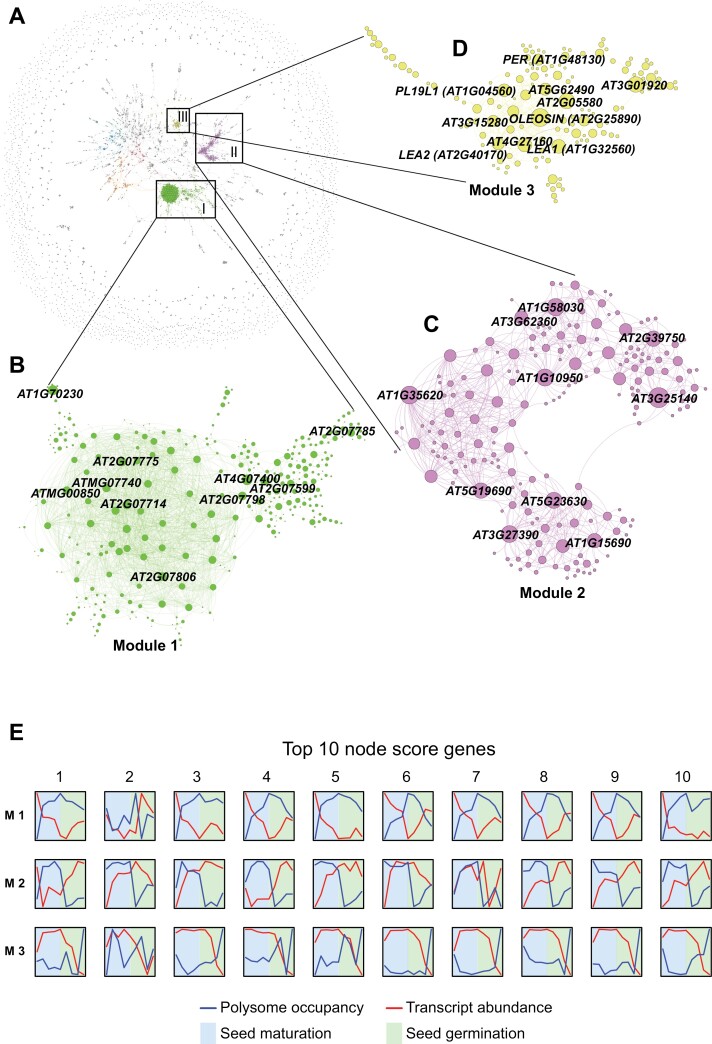
SeedTransNet, a directional gene regulatory inference network based on changes in polysome occupancy (PO) during seed maturation and germination. The data for the network are combined from data presented in this study and the data from Bai *et al.* (2017). (A) SeedTransNet, with nodes representing genes and edges representing pairwise PO linkage. Colour indicates the modules detected from SeedTransNet; the major modules are colour coded. (B–D) Module topology of the top three modules. Nodes with high connectivity within each module are depicted as larger circles. Module 1 includes 334 genes with 1799 gene linkage inferences (GLIs) and an average module node degree of 10.78. Module 2 contains 364 genes and 684 GLIs with an average module node degree of 6.91. Module 3 consists of 140 genes representing 840 GLIs with an average module node degree of 4. (E) Transcript level and PO plotted across seed maturation (blue) and germination (green). The relative levels of the top 10 nodes from each of the top three modules are displayed. Blue lines represent PO and red lines indicate the transcript level in total RNA preparations.

Co-expression cluster analysis describes translational patterns but neither interaction nor directionality, thus we cannot predict regulators or their targets from this analysis. However, because our data represent a time course, they can be used to determine regulatory pathways by means of network analysis. We therefore employed a directed regulatory network approach using the Seidr toolkit (Schiffthaler *et al.*, 2019, Preprint). Seidr is a gene network inference toolkit that implements the core ideas of Marbach *et al.* (2012), namely it utilizes the ‘Wisdom of Crowds’ when inferring gene networks. Briefly, it operates by computing subnetworks of the gene expression data using a variety of published network inference algorithms based on differing statistical principles (correlation, mutual information, and machine learning regression). The networks as implemented in Seidr differ from those inferred by co-expression, as some of the algorithms Seidr utilizes connect two genes if they show either high dependence (not necessarily correlation) or statistical predictability. Algorithms used in Seidr can link patterns that are not exactly co-expressed, since a regulator can be expected to be translated before its target. The output of Seidr is a weighted gene–gene association matrix based on the translational profile including the source and target of every edge, their interaction strengths, directionality (which is either directional or non-directional), and ranks for each of the inference algorithms (Schiffthaler *et al.*, 2019, Preprint). Based on the network topology (measured by its scale-free transitivity and average clustering coefficient), a stringent threshold score was devised to select the most relevant network edges (Supplementary Fig. S4; see the Materials and methods). This resulted in a putative gene regulatory network, which we call the seed translation network (SeedTransNet), with 7873 nodes and 8740 edges, 2298 of which are directional, suggesting regulatory dependence (Fig. 4A; Supplementary Dataset S7). Networks that include highly connected gene nodes can reveal functionally coherent modules and their regulators (Segal *et al.*, 2003). The modules identified in SeedTransNet were organized in a hierarchical modular structure based on the Infomap results (level M1 to M5, Supplementary Dataset S8). Infomap is a way to quantify connectivity by simulating a random walker in the network (Rosvall and Bergström, 2008). Each module level represents a different level of granularity (the first level of granularity depicts the broad picture while the last represents individual connections in the network) which may indicate a functional hierarchy in the network. SeedTransNet is characterized by a highly connected centre core surrounded by a fragmented network ring with relatively low interconnectivity. In total, 1857 modules were identified at the first level of granularity (Module 1), out of which 572 could be assigned a specific biological function through GO enrichment analysis (Supplementary Datasets S8, S9). The top three modules, Modules 1, 2, and 3 (identified at the first granularity level), were further explored to carry out gene linkage inference (GLI) based on high gene linkage prediction (10.5% of the total putative gene translational linkage) (Fig. 4B–D; Supplementary Dataset S8). The high degree of directionality detected indicates that SeedTransNet could be used to discover new translational regulatory pathways.

### SeedTransNet groups translationally co-regulated genes involved in similar processes

Module 1 represents transcripts whose levels were greatly reduced during seed maturation but increased during seed germination, while the opposite dynamics were observed for PO (Fig. 4E). Module 1 is enriched for mRNAs encoding mitochondrial proteins (21%), especially those involved in translation and mitochondrial electron transport (Supplementary Dataset S9), indicating that mRNAs encoding mitochondrial proteins are probably under stringent translational control during seed maturation and germination.

Module 2 contains transcripts that showed high levels of PO during seed maturation, similarly to those in Module 1, but the transcripts dissociated from the polysomes during early seed imbibition and were recruited during the late stage of seed germination, a pattern different from that in Module 1 (Fig. 4E). Gene enrichment analysis identified diverse gene functions including transport and protein glycosylation (Fig. 4C, E; Supplementary Dataset S9). Among the transporters identified was the well-known germination regulator vacuolar Ca^2+^-activated channel protein gene *TPC1*, which governs sensitivity to ABA (a phytohormone that inhibits seed germination) during seed germination and thus the *tpc1* mutant is not responsive to ABA during germination (Peiter *et al.*, 2005). Protein glycosylation is important for cell wall synthesis and maturation, which is also an active process during seed germination due to high levels of cell division and elongation.

Module 3 displayed a low PO during seed maturation while the transcripts accumulated. These accumulated transcripts showed a steep reduction in abundance upon seed germination when the PO drastically increased (Fig. 4E). These transcripts are likely to be seed-stored mRNAs that accumulate in seeds and are translated at different stages during germination (Fig. 4E). GO enrichment analysis showed that genes in this module were associated mainly with seed development, post-embryonic development, seed dormancy and germination, and lipid biogenesis; they included a large number of late embryo abundant (LEA) protein genes, oleosin genes, and seed storage protein genes (Fig. 4D; Supplementary Datasets S9, S10). The LEA proteins were translationally linked with oleosin family proteins, crucifer family seed storage proteins, and other proteins related to ABA, cell wall synthesis, and reactive oxygen species (ROS) balance (Supplementary Fig. S5A). Module 3 was associated with seed maturation because of its enrichment for genes regulated by the *ABSCISIC ACID INSENSITIVE3* (*ABI3*) regulon. *ABI3* is one of the key regulators of seed development (Mönke *et al.*, 2012). A total of 43.9% of the genes in the *ABI3* core regulon were specifically identified in this module (Supplementary Fig. S5B, C; Supplementary Dataset S11), indicating that Module 3 is associated with downstream targets of ABI3. This linkage was connected with genes encoding oleosin, LEA, and storage protein in SeedTransNet (Supplementary Fig. S5C), indicating that these proteins share common features modulated by ABI3. Overall, the network analysis identified putative modules with distinct functions, and this approach could be used for further gene regulatory inference.

### Transcript features correlate with translational regulation during seed maturation

Transcripts that increased in PO had generally longer CDSs, and the opposite was observed for transcripts with reduced PO (Supplementary Fig. S6A). A significantly higher GC content was detected in the CDS region of transcripts with increased PO (Supplementary Fig. S6B). No significant difference was found for GC3 (guanine and cytosine content in the third codon position; synonymous sites) and effective number of codons (Nc) (Supplementary Fig. S6C), indicating that codon bias is not separating the translationally up- or down-regulated mRNAs during seed maturation. To test whether differently regulated mRNAs have different overall mRNA secondary structure, we compared an experimentally derived structure score defined by Li *et al.* (2012) for the different sets of mRNAs. The score is an indicator of potential transcript complexity; high structure scores are equivalent to more double-stranded structures at a certain position in a transcript compared with regions with lower scores. In general, the translationally up-regulated genes had lower than average structure scores (Supplementary Fig. S6D), especially in the coding region. Translationally down-regulated genes in this analysis showed higher than average scores, especially at the end of the 5ʹ leaders and in the coding region. The transcripts with the highest structure scores were enriched for photosynthesis and thylakoid GO categories (Supplementary Dataset S12). This may suggest that the translational regulation of these photosynthesis-related genes during seed maturation is mediated by a mechanism involving the mRNA secondary structure.

Translational regulation may be mediated through *cis*-elements in target mRNAs. By comparing mRNA sequences in each module with mRNA sequences from the whole genome, motifs specifically enriched in different regions for each module were identified (Supplementary Fig. S7). Pyrimidine- (UC) enriched motifs were identified in the 5ʹUTR in Module 1, while motifs enriched in pyrimidine with different repeats (UCM and CUU) and complementary purine repeats (GA and GAA) were identified in the 5ʹUTR and cDNA sequences in Module 2. In Module 3, an A-rich motif, AACAAAAAA, was enriched in the 5ʹUTR (Supplementary Fig. S7). Interestingly, the same motif was identified in transcripts that showed increased PO during early seed germination (Bai *et al.*, 2017). A similar poly(A) motif was recently identified as playing a role in gene-specific translational regulation in response to pathogens by interacting with poly(A)-binding proteins (Xu *et al.*, 2017). Overall different motifs are enriched in different sections of the network and can therefore be seen as supporting the biological relevance of the network.

### Network inference successfully predicts translational regulatory circuits

The dependency-based property of SeedTransNet allows the existence of directional gene interactions to be inferred. In total, 65 directed gene inferences were made, all in the three modules with the highest modularity ranking, among which 43 were in Module 3 (Supplementary Dataset S10). To select the nodes that may constitute the main translational regulators and avoid false predictions, an arbitrary node degree cut-off of 4 was used. Five nodes passed this selection criterion. These five nodes, which were associated with embryo maturation, were 1-cysteine peroxiredoxin1 (*PER1*, *AT1G48130*), *AWPM-19-LIKE* protein (*AT1G04560*), *CRUCIFERINA1* (*CRA1*, *AT5G44120*), *CRUCIFERIN2* (*CRU2*, *AT1G03880*), and *CRUCIFERIN3* (*CRU3*, *AT4G28520*).

**Fig. 5.**
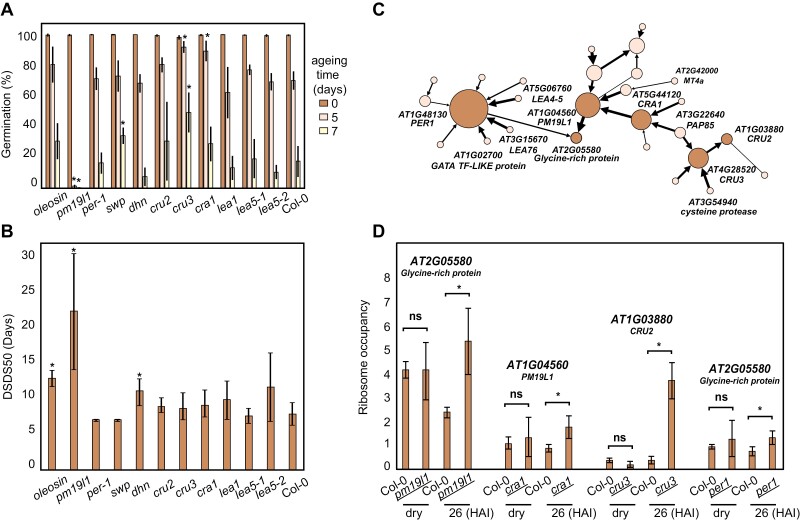
SeedTransNet regulatory network hub genes affect seed characteristics. (A) Seed longevity phenotypes for plants with mutations in hub genes selected from Module 3. Five and seven days of artificial ageing were used to evaluate seed longevity in comparison with zero days of ageing. Error bars indicate average ±SD (*n*=4); asterisks indicate *P*-value <0.05 (Student’s *t*-test) as compared with Col-0 seeds. (B) Seed dormancy phenotypes of plants with mutations in hub genes selected from Module 3 represented by DSDS50 (the number of days of seed dry storage required to reach 50% germination). Error bars indicate average ±SD (*n*=4); asterisks indicate *P*-value <0.05 (Student’s *t*-test). (C) Close-up of a subset of the directional network in Module 3, centred around *PM19L1*, *AT1G04560*, and the target gene *AT2G05580*, *Glycine-Rich Protein*, which was confirmed experimentally (both red). Strengths of the predictions are indicated by edge thickness. (D) Ribosome occupancy (RO, ribosomal mRNA/total mRNA) of the downstream transcript targets (bold italic) of the predicted nodes from (C) is different in response to imbibition in wild-type and in mutant seeds (underlined) of the respective upstream nodes. Error bars indicate the average ±SD (*n*=3), *P*-value <0.05 (*t*-test).

For M3, we tested the influence of these network hub nodes, in both a directional and a non-directional way, on seed maturation and germination by investigating their seed dormancy and longevity phenotypes. A large proportion of the mutants tested showed seed-related phenotypes (Fig. 5; Supplementary Fig. S8). One of the hub node genes encoded the *AWPM-19-LIKE1* (referred to here as *PM19L1*) protein, and seeds of plants with this gene mutated showed an extreme sensitivity to ageing, as perceived from the severe reduction in seed germination after 5 d of artificial ageing compared with 70% germination at the same ageing stage of wild-type seeds. Similar results were found for two independent T-DNA knockout mutants, and the artificial ageing sensitivity could be complemented by overexpression of *PM19L1* driven by the 35S promoter (Fig. 5A; Supplementary Fig. S8). The mutant *pm1911* also showed significantly enhanced seed dormancy (Fig. 5B), as was also shown independently by Barrero *et al.* (2019). Taken together, these findings indicate that the PM19L1 protein plays an essential role in seed physiology.

Judging from SeedTransNet inference, PM19L1, PER1, CRU1, and CRU3 were predicted as translational regulators (Fig. 5C). PM19L1 regulates the translation of the downstream target gene *AT2G05580*, which encodes a glycine-rich protein (Fig. 5C). Public databases of the protein–protein interaction network STRING (Szklarczyk *et al.*, 2015) and the co-expression network GeneMANIA (Warde-Farley *et al.*, 2010) did not predict direct co-expression for the two genes, although they share some co-expressed gene nodes (Supplementary Fig. 8A, B) such as LEA protein genes and oleosin family genes. The expression of both of the *PM19L1* and *Glycine-Rich Protein* genes is highly seed specific (Supplementary Fig. S9C, D). We hypothesized that PM19L1 may translationally regulate the *AT2G05580* gene. To validate this, ribosome-associated mRNAs were isolated from fully after-ripened (non-dormant) *pm19l1* and Col-0 seeds, both dry and 26 h after imbibition. Upon germination, the polysomal occupancy of *AT2G05580* was >2-fold decreased in the Col-0 seeds (Fig. 5D). This was abolished in the *pm19l1* mutant, indicating that SeedTransNet can predict putative regulatory relationships. Similar disruption of polysomal occupancy was also seen in *per1*, *cru1*, and *cru3* mutants (Fig. 5D). The *pm19l* mutation is affecting the ribosomal association of *AT2G05580* without affecting the transcript levels, while mutations in *PER1*, *CRU1*, and *CRU3* are affecting both transcript levels and ribosomal association of their respective target mRNAs (Supplementary Fig. S10). This validates and indicates the power of SeedTransNet for regulatory pathway prediction.

## Discussion

Seed maturation is the developmental process that starts when the embryo is fully developed and ends with the completely dried seed. Maturation is characterized by growth arrest, storage reserve accumulation, acquisition of desiccation tolerance, and the induction of seed dormancy and seed longevity. This developmental programme is under the control of the key regulator ABI3 (Koornneef *et al.*, 1982; Ooms *et al.*, 1993; Mönke *et al.*, 2012; Baud *et al.*, 2016). Here we show that seed maturation is associated with global translational changes. These changes are developmentally regulated and not simply a consequence of drying, as the translational change is absent in dry seeds of *abi3-5*. We therefore set out to characterize the translational changes at the gene level and showed that translational events during seed maturation are linked to similar events during germination and seedling establishment (Bai *et al.*, 2017). As is the case for previously described events of translational reprogramming, multiple regulatory pathways operate in concert. This underlines our current lack of understanding of translational regulation in plants and means that our work is a stepping-stone towards gaining a greater understanding of gene regulation from gene sequence to synthesized protein.

### Translational reprogramming affects the expression of seed maturation genes

Genome-wide polysome profiling indicated that translational regulation is concentrated during the early phase of seed maturation (maturation translational shift, MTS) from 12 to 15 DAF. The MTS occurs at a stage when there are no obvious changes in cellular processes; cell expansion, cotyledon and axis development, and reserve accumulation are still occurring. The MTS may therefore precede developmental changes rather than reflecting changes that have already happened. Many of the MTS genes are also transitionally regulated at later stages and are present among the HTS and GTS genes that were previously identified during seed germination (Bai *et al.*, 2017). Many of the genes that are translationally regulated at multiple time points are related to seed desiccation, such as some encoding dehydrins and heat shock proteins (Supplementary Dataset S2). A similar desiccation-dependent mechanism may also operate in leaves, as the seed-specific LEA protein (LEA14, AT1G01470), heat shock protein (HSP70, AT3G12580), and desiccation-responsive protein (RD29A, AT5G52310) gene transcripts are also translationally regulated in leaf tissue in response to dehydration (Kawaguchi *et al.*, 2004).

MTS genes display diverse transcript levels and translational profiles, which suggests that different regulatory mechanisms operate in parallel (Supplementary Fig. S11). Among the translationally controlled genes are some that have been reported as seed-specific regulators, such as *ABSCISIC ACID-INSENSITIVE 2* and *5* (*ABI2* and *ABI5*) (Supplementary Dataset S11), mutations in which result in insensitivity to ABA during seed germination (Finkelstein, 1994; Rodriguez *et al.*, 1998; Finkelstein and Lynch, 2000). The translational profiles of both *ABI2* and *ABI5* mRNAs correlate with the pattern of ABA accumulation during seed maturation, which peaks during early seed maturation when cell elongation is terminated (Vicente-Carbajosa and Carbonero, 2005). The *STAY-GREEN2* (*SGR2*) gene is important for regulating the chlorophyll content during seed maturation and it is also translationally regulated. The MTS also contains a gene involved in seed dormancy, seed dormancy regulator *REDUCED DORMANCY 4* (*RDO4*) (Liu *et al.*, 2007). These examples imply a role for translational control of molecular events preparing seeds for their final maturation and priming the seed for germination.

### Network analysis reveals co-regulated mRNAs

Gene network inference analysis allows prediction of the functions of uncharacterized genes using the guilt-by-association principle (Wolfe *et al.*, 2005), which postulates that genes sharing a similar expression profile tend to have similar functions or be involved in the same molecular pathways or functions. This strategy has been widely applied in different biological contexts and has facilitated the generation of hypotheses about gene functions and gene–gene associations (Angelovici *et al.*, 2009; Bassel *et al.*, 2011; Verdier *et al.*, 2013; Costa *et al.*, 2015; Silva *et al.*, 2016). However, such clustering approaches disregard edge directionality as well as non-linearity of the regulatory cascades, and are susceptible to overfitting, resulting in high network complexities; hence they are unsuitable for network reconstruction, at least without additional assumptions (Margolin *et al.*, 2006). The construction of gene regulatory networks is generally based on calculation of the pairwise confidence scores of every edge between gene pairs (the nodes), followed by a score-based selection of edges within the full network to infer its topology, resulting in a putative causal network. More recently, Marbach *et al.* (2012) demonstrated that aggregating several gene network inferences (GNIs) in a single meta-network outperformed every individual GNI; this is the so-called ‘crowd knowledge’. This approach has recently been implemented in the Seidr software package (Schiffthaler *et al.*, 2019, Preprint).

Seed-specific network analysis has been performed to identify genes that play roles in seed dormancy, longevity, and seed–seedling transition (Bassel *et al.*, 2011; Verdier *et al.*, 2013; Righetti *et al.*, 2015; Silva *et al.*, 2016). However, none of these networks is directional and therefore they cannot be directly used in understanding regulatory circuits. By constructing a directional co-translational network, SeedTransNet, with data from consecutive developmental phases including both seed maturation and germination, we could identify functional modules with distinct functions along the whole time course. Using this network, we were able to predict gene regulatory hubs and directional regulatory cascades, later proved by experimental confirmation.

To first evaluate SeedTransNet, we undertook GO analysis and confirmed that the different modules clustered genes with separate functions. The modules also contain genes harbouring distinct sequence motifs, consistent with separate regulatory patterns as defined by the network. Although only PO data (ratios of mRNA levels in the polysomal pool to total RNA) were used to generate the network, it predicts the individual changes in gene transcript levels as well indicating that transcript and translational patterns are regulated in concert. Finally, we were able to prove that several of the node genes identified encode factors that affect seed-specific phenotypes by studying the effects of mutations in these genes.

The large-scale structure of the network indicates the existence of three major modules. Module 1 is enriched with translational factors including substantial numbers of rRNAs and tRNAs, corresponding to the dramatic changes in ribosome profile identified during seed maturation and germination (Fig. 1A; Bai *et al.*, 2017). The modulization of both translational components may indicate coordinated regulation to shape the translational programme during both seed maturation and germination. Oxidation–reduction processes are identified as important in the network analysis (Supplementary Dataset S9). Low-oxygen stress during maturation could lead to an accumulation of electrons from the inner mitochondrial membrane, resulting in ROS production (Patel *et al.*, 2019). ROS in seeds are important signalling molecules for breaking seed dormancy, while excess accumulation of ROS could trigger ageing processes leading to seed deterioration and death (El-Maarouf-Bouteau and Bailly, 2008; Bailly and Kranner, 2011; Leymarie *et al.*, 2012).

Several genes in Module 2 encode transporters, which are involved in transporting different ions such as calcium and manganese (ECA3, AT1G10130), nitrate (NRT1.1, AT1G12110), sodium, potassium, and chloride (CCC1, AT1G30450), and small molecules such as purine (AT1G21070), phytochelatin and arsenite (ABCC1, AT1G30400), and xenobiotic transporters (ABCC5, AT1G04120). These transporter genes show an increase in transcript level during seed maturation while the PO values for these genes are constant, indicating extensive metabolic exchange during seed reserve filling, which provides the essential substrates for early seed germination. However, the specific functions of these translationally associated transporter genes remain to be revealed.

Module 3 contains a large number of proteins well known to be important for seed maturation, such as LEA proteins, oleosin proteins, and proteins involved in ABI3 cascades, strongly indicating translational coordination between the proteins translated during seed maturation. Specifically, Module 3 contains genes with high transcript abundance during maturation (Fig. 4E), which are therefore likely to represent seed-stored mRNAs. As recently reported, seed-stored mRNAs are important for seed germination, being translated at an early stage during seed germination (Bai *et al.*, 2020). This is also reflected by the PO, which is relatively low during maturation but dramatically enhanced during germination. A delay in the translation of LEA proteins has been reported previously; some LEAs appear hours or even days after transcription of their genes (Galau *et al.*, 1987; Hughes and Galau, 1989; Espelund *et al.*, 1992; Chatelain *et al.*, 2012; Verdier *et al.*, 2013). The translational regulation of these protective proteins provides seeds with an adaptive mechanism to protect the cellular status during phases of de- and rehydration.

In order to facilitate the investigation of the network, we have made a web-based interface with which we can search for genes, plot local network environments, and overlay the subnetwork with auxiliary information such as GO data, protein domain information, presence of defined sequence elements, etc. This served as a tool for our investigations but will also benefit other researchers (available on http://seedtransnet.plantgenie.org/).

### Using SeedTransNet to unravel regulatory pathways

We explore the function of one of the central hubs identified from SeedTransNet named *PM19L1*, which was shown to regulate seed dormancy by Barrero *et al.* (2019). We confirm the data from Barrero *et al.* (2019) and show that the gene is also important for seed longevity (Fig. 5A). The high *PM19L1* transcript level specifically in dry seeds and its connection with other seed maturation genes indicates its importance during seed maturation (Fig. 5C; Supplementary Fig. S8). The PM19L1 homologue was originally identified in wheat (*Triticum aestivum*) as AWPM-19 (ABA-induced wheat plasma membrane polypeptide-19), which accumulates during the development of ABA-induced freezing tolerance (Koike *et al.*, 1997). Recently, a rice homologue, OsPM1, has been identified as being associated with ABA-induced drought tolerance and seed germination rate (Yao *et al.*, 2018). With SeedTransNet, we were able to further validate its downstream target *AT2G05580. PM19L1* influences the ribosome association and probably the translation of *AT2G05580*. We also validated other translational regulatory inferences from SeedTransNet such as CRA1–*PM19L1*, CRU3–*CRU2*, and PER1–*AT2G05580* (Fig. 5C). Mutations in the predicted regulators all affect the ribosomal occupancy of the predicted targets. The prediction is based on polysomal occupancy changes which in many cases is viewed as a proxy of translational activity. Protein levels are determined by more factors including protein degradation. Moreover, the predictions do not imply direct regulatory control. In these cases, direct regulatory interaction is less likely since the predicted regulators are seed storage proteins. The role of these storage proteins in translational control is unknown, but the abundance of CRU2 is probably linked to that of CRU3 while independent of the abundance of CRA1, as shown by SeedTransNet (Fig. 5C). As inferred from SeedTransNet, CRU3 strongly regulates the translation of *CRU2* (Fig. 5C, D). This is possibly caused by co-localized translation of CRU2 and CRU3 leading to the dependency of CRU2 translation on CRU3, similar to the seed storage proteins prolamine and glutelin in rice (Withana-Gamage *et al.*, 2013; Tian *et al.*, 2020).

**Fig. 6.**
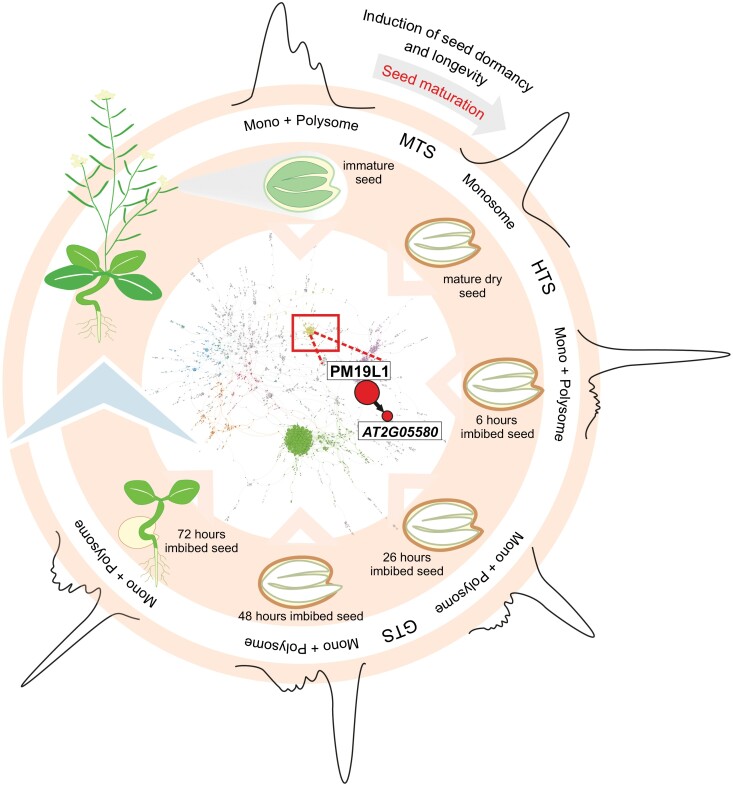
Model summarizing the results of this study. The centre shows the seed translational network (SeedTransNet) that was built based on the polysome occupancy (PO) data obtained after polysome analysis at six stages from seed maturation (four time points) until seedling establishment (five time points). The different developmental stages are presented in the inner ring, and the polysome profiles on the outer ring. The red square indicates the seed maturation module, with a zoom in to PM19L1, a key regulator identified in the network that affects the translation of a glycine-rich transcript. Seed dormancy and longevity are determined during seed maturation; regulators of seed maturation therefore often contribute to dormancy and longevity phenotypes. This is also reflected in the phenotype of the *pm19l1* mutant seeds (Fig. 5).

Overall, mRNA translation during seed maturation is extensively modulated using several different molecular mechanisms. This study reveals substantial translational dynamics during the early phases of plant development, summarized in Fig. 6. Our network is a valuable tool for identifying seed-specific regulation mechanisms, which may be suitable for further scientific investigations or as possible breeding targets for improving seed characteristics. To facilitate the work of other researchers, we have made the network public and easily accessible so that researchers with an interest in seed biology but lacking the computational skills needed to mine network data can investigate translational regulation of their favourite gene or physiological process. In summary, our analysis has further emphasized the importance of translational regulation and *cis-*regulatory motifs in mRNAs. There is a universe of regulatory potential that has not yet been investigated. In the coming years, more plant and seed researchers will turn their attention towards translational biology.

## Supplementary data

The following supplementary data are available at *JXB* online.

Fig. S1. Summary of statistical processing of microarray data.

Fig. S2. Comparison of the transcriptional changes during seed maturation as documented in this manuscript with previously published data.

Fig. S3. Comparison of translationally regulated genes during seed maturation with another set of translationally regulated genes.

Fig. S4. Threshold selection for SeedTransNet construction.

Fig. S5. Comparison between regulons identified in SeedTransNet and previously published seed maturation regulons.

Fig. S6. Sequence features of genes translationally regulated during seed maturation.

Fig. S7. Sequence motifs enriched in SeedTransNet mRNAs.

Fig. S8. Complementation of *atpm1* seed longevity phenotype by overexpression of the *PM19L1* gene.

Fig. S9. Network database comparisons for genes predicted from SeedTransNet.

Fig. S10. Ribosomal occupancy changes of target genes determined by SeedTransNet.

Fig. S11. Intensity profiles of genes with changed PO.

Table S1. Primers and vectors used in this study.

Table S2. Transgenes and mutants used in this study.

Dataset S1. Raw normalized intensity levels for total RNA (T) and polysomal RNA (P) (log_2_ transformed) during seed maturation.

Dataset S2. Genes translationally regulated during seed maturation.

Dataset S3. GO enrichment analysis of genes translationally regulated during seed maturation.

Dataset S4. Raw normalized intensity levels for total RNA (T) and polysomal RNA (P) (log_2_ transformed) for the combined dataset (maturation and germination).

Dataset S5. Transcript clusters based on the dynamics in PO during seed maturation and germination.

Dataset S6. GO enrichment analysis of the genes in the individual clusters from hierarchical clustering of PO time course during seed maturation and germination (Fig. 3).

Dataset S7. Seed translation network (SeedTransNet). Columns listed are resource, target, linkage weight, and direction for the network.

Dataset S8. Infomap community prediction and gene linkage inference by SeedTransNet.

Dataset S9. GO enrichment analysis of genes in SeedTransNet modules.

Dataset S10. Genes included in the LEA protein network as identified in Module 3 of SeedTransNet.

Dataset S11. Genes included in the ABI3 core regulon network as identified in Module 3 of SeedTransNet.

Dataset S12. GO terms enriched among the 100 genes with the highest structure scores (within the CDS) of the genes with PO during the seed maturation shift.

Dataset S13. Raw normalized signals in the maturation dataset of the known seed maturation-related genes.

Dataset S14. Raw data used for figures.

erac394_suppl_Supplementary_Datasets_S1-S14

erac394_suppl_Supplementary_Figures_S1-S11_Tables_S1-S2

## Data Availability

Raw data are freely available at Gene Expression Omnibus (GEO) repository (http://www.ncbi.nlm.nih.gov/geo/) under accession numbers GSE65780 (germination and seedling establishment) and GSE127509 (seed developmental and maturation). SeedTransNet can be accessed, searched, and investigated at: http://seedtransnet.plantgenie.org/
