# Supplementary material for: SeedTransNet: a directional translational network revealing regulatory patterns during seed maturation and germination
Source: J Exp Bot. 2022 Oct 8;74(7):2416–32. doi: 10.1093/jxb/erac394 (PMC10082931; doi:10.1093/jxb/erac394)
Supplement: erac394_suppl_Supplementary_Figures_S1-S11_Tables_S1-S2 [file erac394_suppl_supplementary_figures_s1-s11_tables_s1-s2.pdf]

Supplementary Information for

Title: SeedTransNet: A directional translational network revealing regulatory patterns during seed maturation and germination

Authors: Bing Bai[a,b#], Bastian Schiffthaler[c], Sjors van der Horst[d], Leo Willems[b], Alexander Vergara[c], Jacob Karström[a], Niklas Mähler[a], Nicolas Delhomme[c], Leónie Bentsink[b]†, Johannes Hanson[a]†*
[a] Umeå Plant Science Center, Department of Plant Physiology, Umeå University, SE-901 87 Umeå, Sweden
[b] Wageningen Seed Science Centre, Laboratory of Plant Physiology, Wageningen University, 6708 PB Wageningen, The Netherlands
[c] Umeå Plant Science Centre, Department of Forest Genetics and Plant Physiology, Swedish University of Agricultural Sciences, Umeå, Sweden
[d] Department of Molecular Plant Physiology, Utrecht University, 3584 CH Utrecht, The Netherlands

[#]Current address: University of Copenhagen, Department of Biology, Ole Maaløes Vej 5, DK-2200, Copenhagen, Denmark

† Last authorship is equally shared

* Corresponding author: johannes.hanson@umu.se.

This PDF file includes:

Supplementary Text
Supplementary Figures S1 to S11
Supplementary Tables S1 and S2

The Supplementary Data Sets are available in a separate excel file.

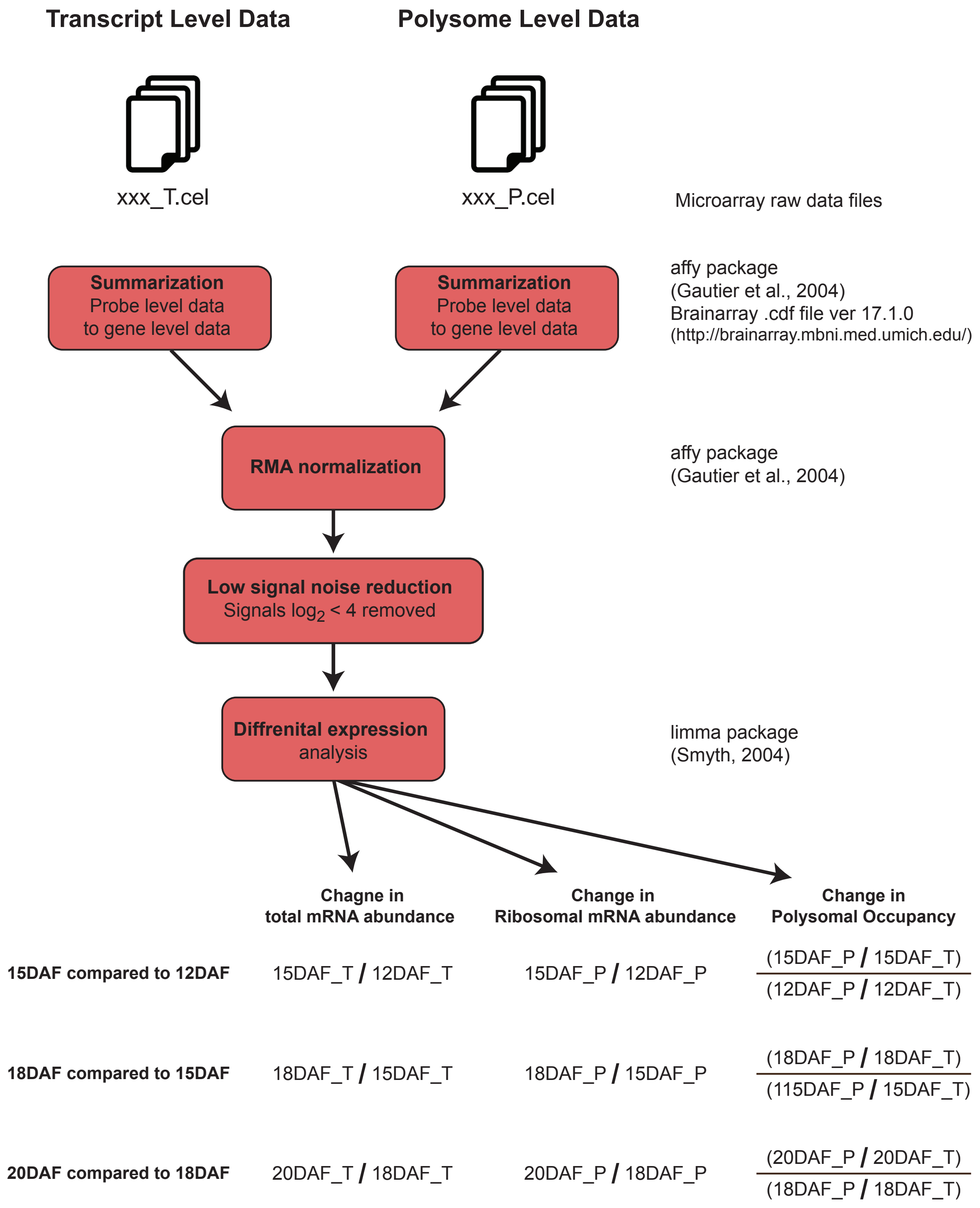

Supplementary Figure S1. Summary of statistical processing of microarray data.

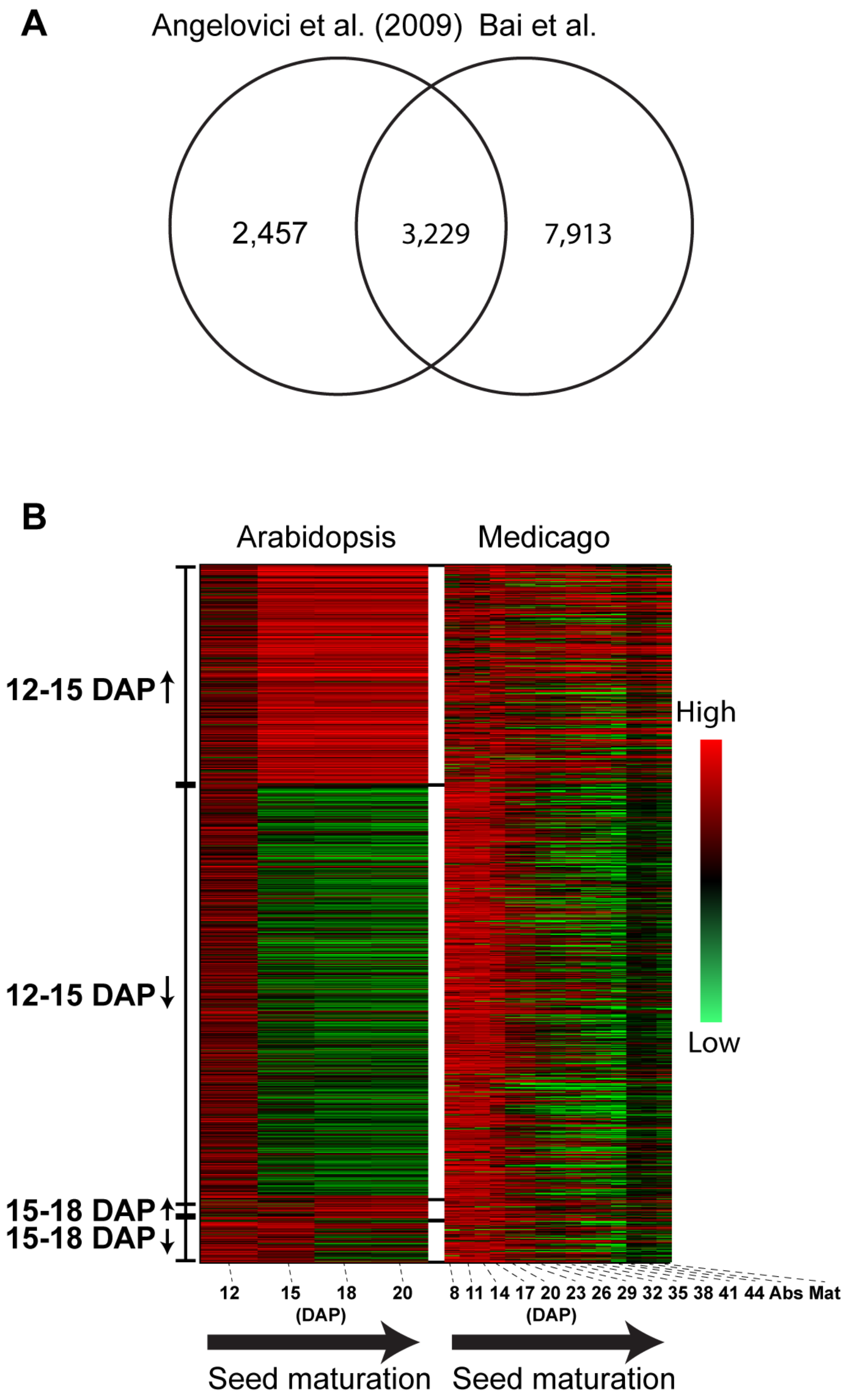

Supplementary Figure S2. Comparison of the transcriptional changes during seed maturation as documented in this manuscript with previously published data.
A. Comparison of genes differentially transcribed from 12 to 20 DAF during Arabidopsis seed maturation identified in the current study with the previously reported to be differentially expressed from 14 DAF to the final mature seed (Angelovici et al., 2009).
B. Heatmap visualization of conserved gene expression profile during seed maturation in both Arabidopsis in the current study and Medicago as reported previously (Righetti et al., 2015). Genes are grouped as up-/down-regulated between 12 to 15 DAF and 15-18 DAF during Arabidopsis seed maturation. Colors indicate relative expression levels from green (low) to red (high).

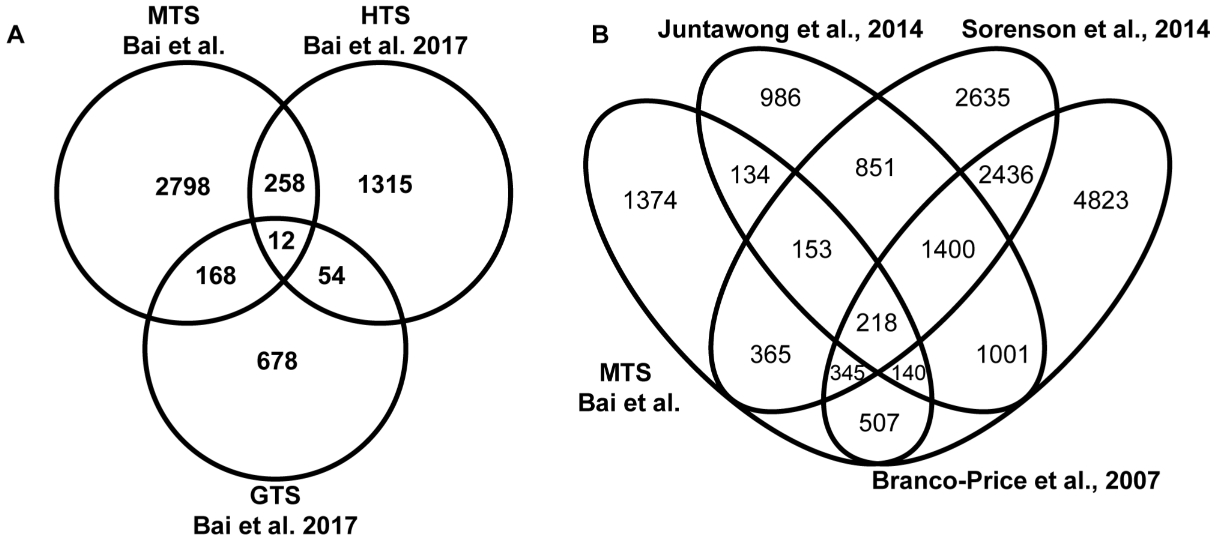

Supplementary Figure S3. Comparison of translationally regulated genes during seed maturation with other set of translationally regulated genes.

A. Comparison of translationally regulated gene during seed maturation translational shift genes (MTS) to the translationally regulated genes during seed germination (Bai et al. 2017).
B. The comparison between the MTS genes in the current study with three translationally regulated gene sets from hypoxia stress (Branco-Price et al., 2005; Juntawong and Bailey-Serres, 2012; Sorenson and Bailey-Serres, 2014)

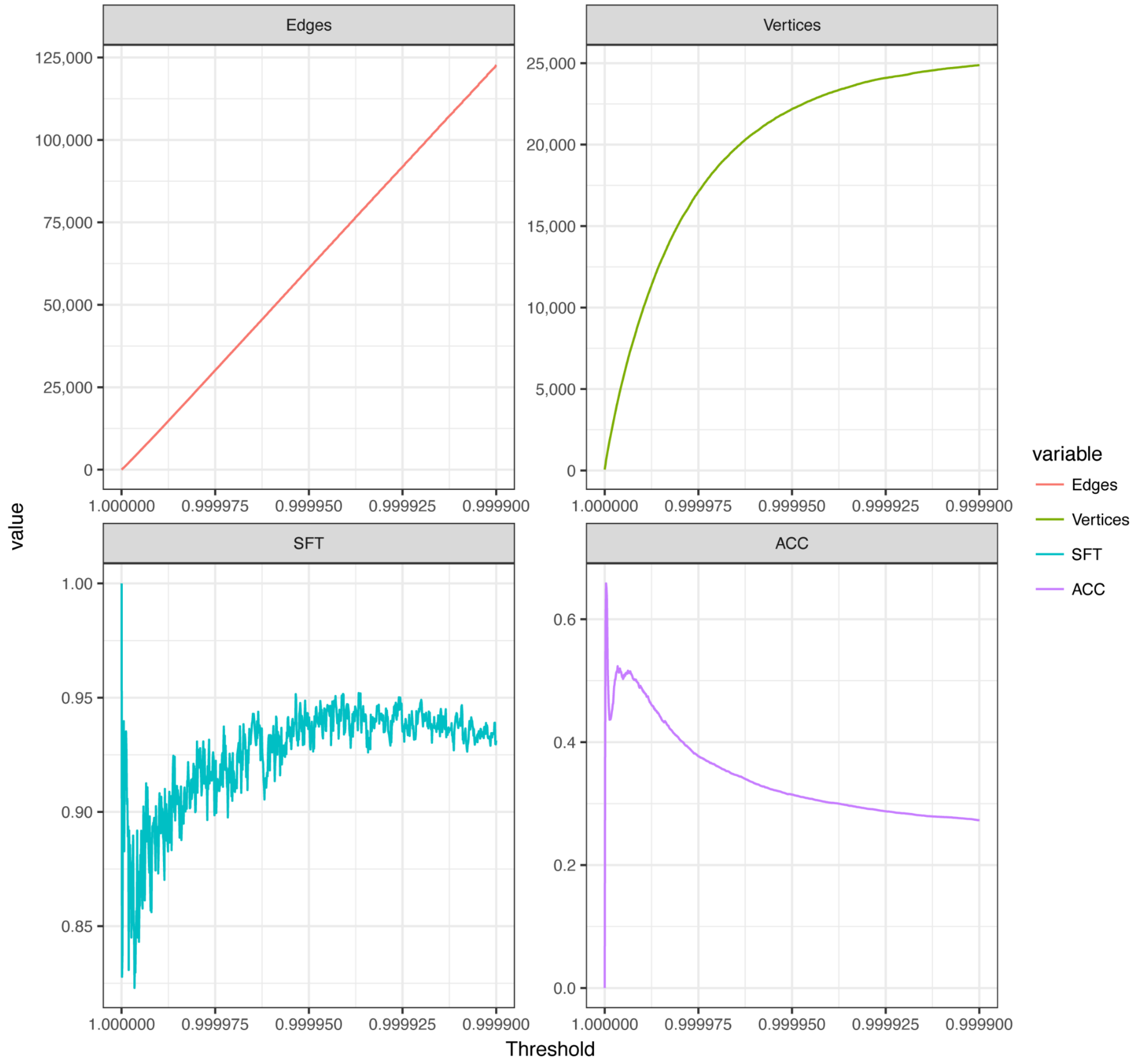

Supplementary Figure S4. Threshold selection for SeedTransNet construction.

The network edges, vertices (nodes), scale free fit (SFT) and transitivity (ACC) are plotted against the network linkage strength calculated by infomap algorithm for cutoff strength selection. A final network linkage strength of 0.999985 is used for final network construction.

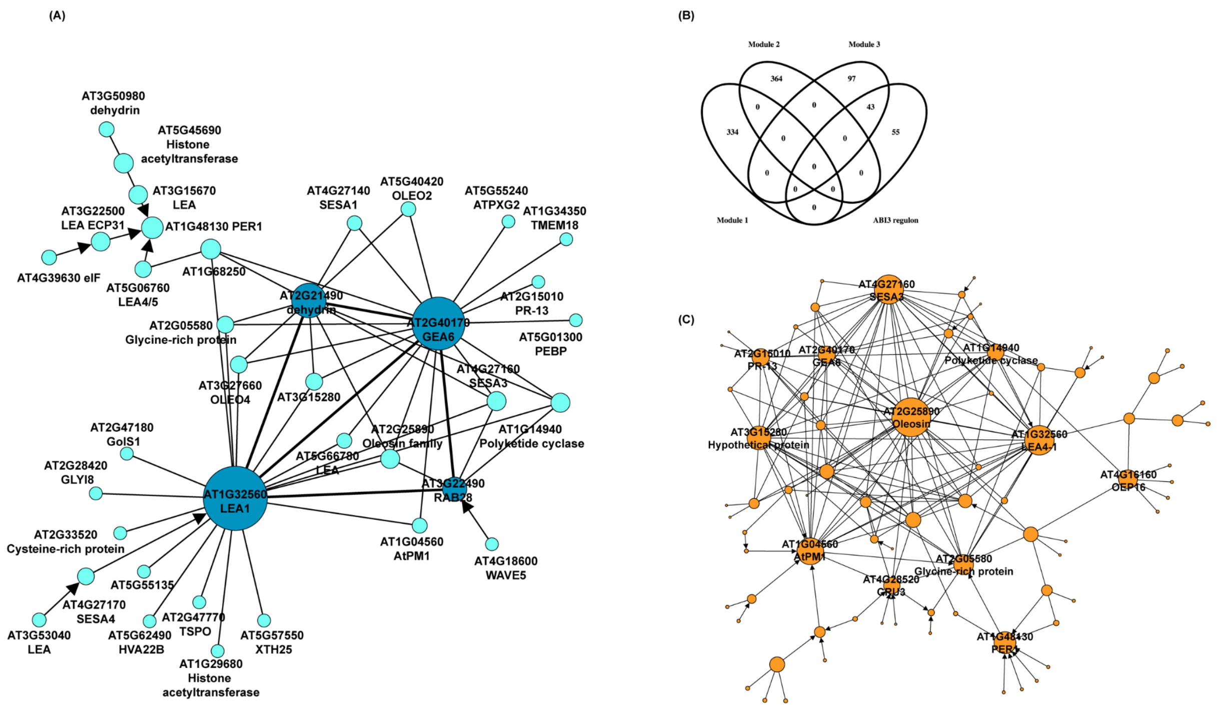

Supplementary Figure S5. Comparison between regulons identified in SeedTransNet and previous published seed maturation regulons.

A. LEA protein network identified in the Module 3. Node size indicates the node connectivity in the network. The thickness of the edges indicates the strength of the node connections. The arrows of the edge indicate the predicted directions. The center highlighted nodes are the four nodes with highest connectivity in the network.
B. Comparison between the ABI3 regulon that are specifically detected in the M1-3 modules with the total previously identified genes in the ABI3 regulon (Monke et al., 2012).
C. ABI3 core regulon network as predicted in the SeedTransNet. Node size indicates the node connectivity in the network. The thickness of the edges indicates the strength of the node connections. The arrows of the edge indicate the predicted directions.

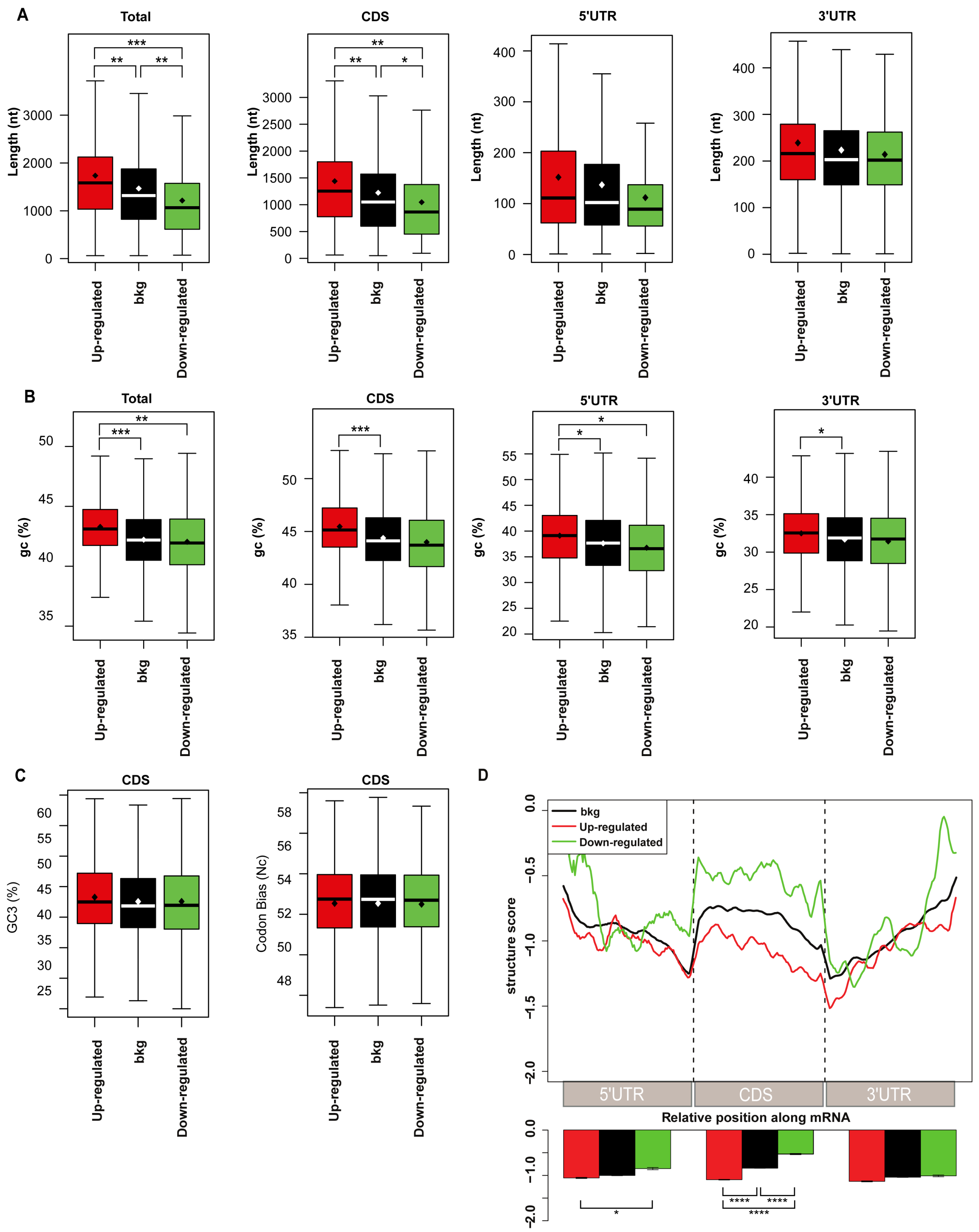

Supplementary Figure S6. Sequence features of genes translational regulated during seed maturation.

A. Lengths of different sequence regions including total mRNA (Total), coding region (CDS), 5'UTR and 3'UTR of the Maturation Translational Shift genes (significantly changed polysomal occupancy PO between 12-15 DAF). Black, all mRNAs expressed in the experiment as background (bkg); red, translationally up-regulated genes; green, translationally down-

regulated genes. Error bars ± SE (*P-value<E-10, ** P-value<E-20, ***P-value<E-50. *P*-values are calculated by a Wilcoxon non-parametric test.

B. GC content in different sequence regions including total mRNA (Total), coding region (CDS), 5'UTR and 3'UTR of the Maturation Translational Shift genes (significantly changed polysomal occupancy PO between 12-15 DAF). Black, all mRNAs expressed in the experiment as background (bkg); red, translationally up-regulated genes; green, translationally down-regulated genes. Error bars ± SE (*P-value<E-10, ** P-value<E-20, ***P-value<E-50. *P*-values are calculated by a Wilcoxon non-parametric test.

C. GC3 content and codon bias (Nc) of coding region (CDS) of the Maturation Translational Shift genes (significantly changed polysomal occupancy PO between 12-15 DAF). Black, all mRNAs expressed in the experiment as background (Bkg); red, translationally up-regulated genes; green, translationally down-regulated genes. Error bars ± SE. P-values are calculated by a Wilcoxon non-parametric test.

D. Secondary structures correlate with polysome occupancy changes. The average structure score is plotted over the 5'UTR, CDS and 3'UTR of the Maturation Translational Shift genes (significantly changed polysomal occupancy PO between 12-15 DAF); black microarray background (bkg); red, genes with increased PO; green, genes with decreased PO. Background; all transcripts on the array (black line), translational up (red line) or translational down transcripts (green line). P-values are calculated by a Student's test according to (Li et al., 2012).

Module	Motif type	Motif logo	Region	FC	*P*-value
1	Pyrimidine	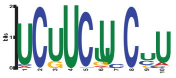	5'UTR	1.54	3.36E-03
2	Pyrimidine	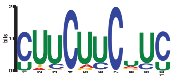	5'UTR	1.43	1.57E-04
		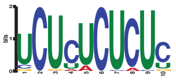	5'UTR	1.71	6.08E-08
		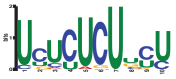	cDNA	1.50	8.32E-25
		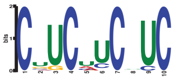	CDS	1.53	1.61E-19
		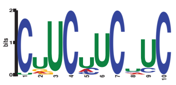	cDNA	1.39	7.06E-17
		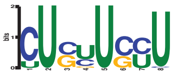	CDS	1.61	2.13E-19
2	Purine	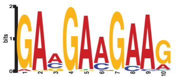	5'UTR	1.41	2.72E-03
		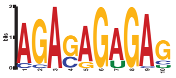	5'UTR	1.23	7.25E-02
		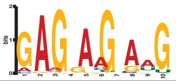	cDNA	1.21	1.85E-06
3	Poly(A)	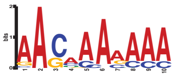	5'UTR	1.87	3.12E-04

Supplementary Figure S7. Sequence motifs enriched in SeedTransNet mRNAs.

The significantly enriched motifs detected in Modules 1, 2 and 3 of SeedTransNet. The sequence logo, motif localization, fold change (FC) enrichment compared to the background sequence (total microarray background sequence for each sequence region) and *P*-value (Fisher test) are shown.

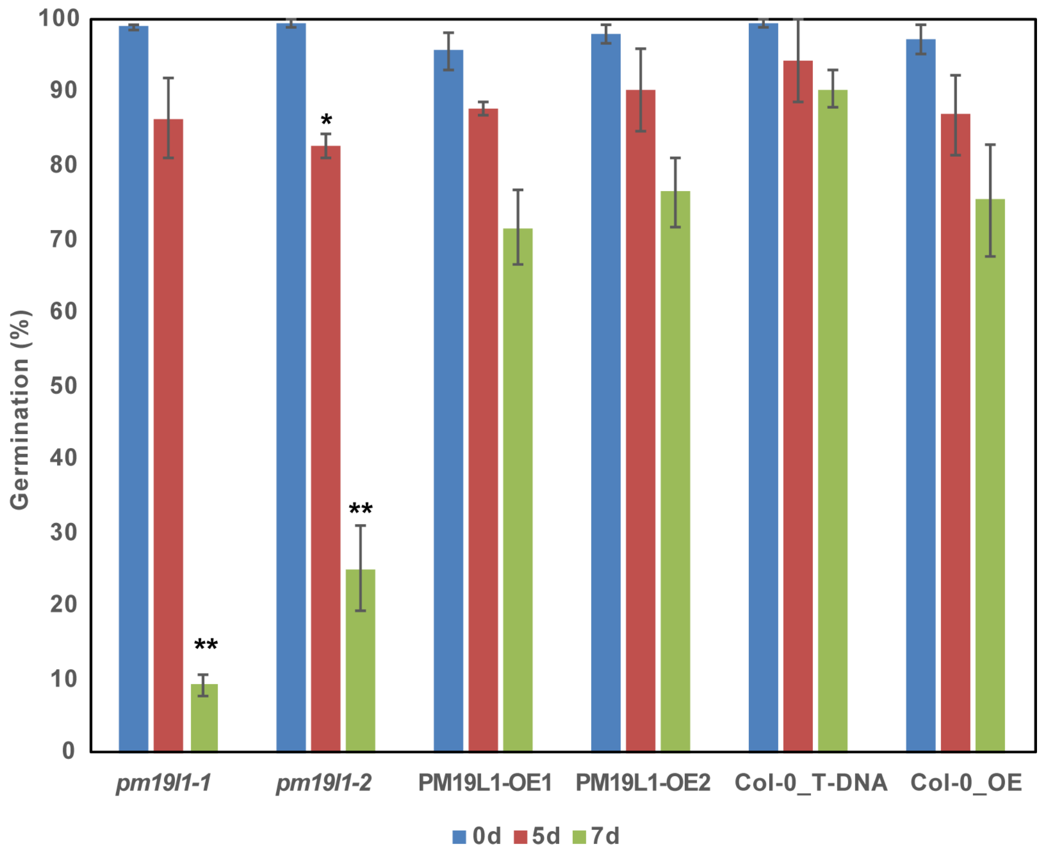

Supplementary Figure S8. Complementation of *atpm1* seed longevity phenotype by overexpression of the *PM19L1* gene.

Two independent T-DNA mutants of PM19L1, *pm19l1-1* and *pm19l1-2* (SALK_075435 and SALK_062287) are used for phenotyping in this experiment. Two different overexpression lined with 35S promoter PM19L1-OE1 and PM19L1-OE2 are used to complement the *pm19l1* (SALK_075435). Seed longevity of mutant and complementation transgenes are compared between Col-0 wt plants. Results from corresponding and parallelly grown wt stocks for both PM19L1 mutants (Col-0_T-DNA) and PLM19L1 overexpressors (Col-0_OE) are shown separately. Error bars indicate average ±SD (n= 4), stars indicate *P*-value (*< 0.05, **< 0.01; Students t-test).

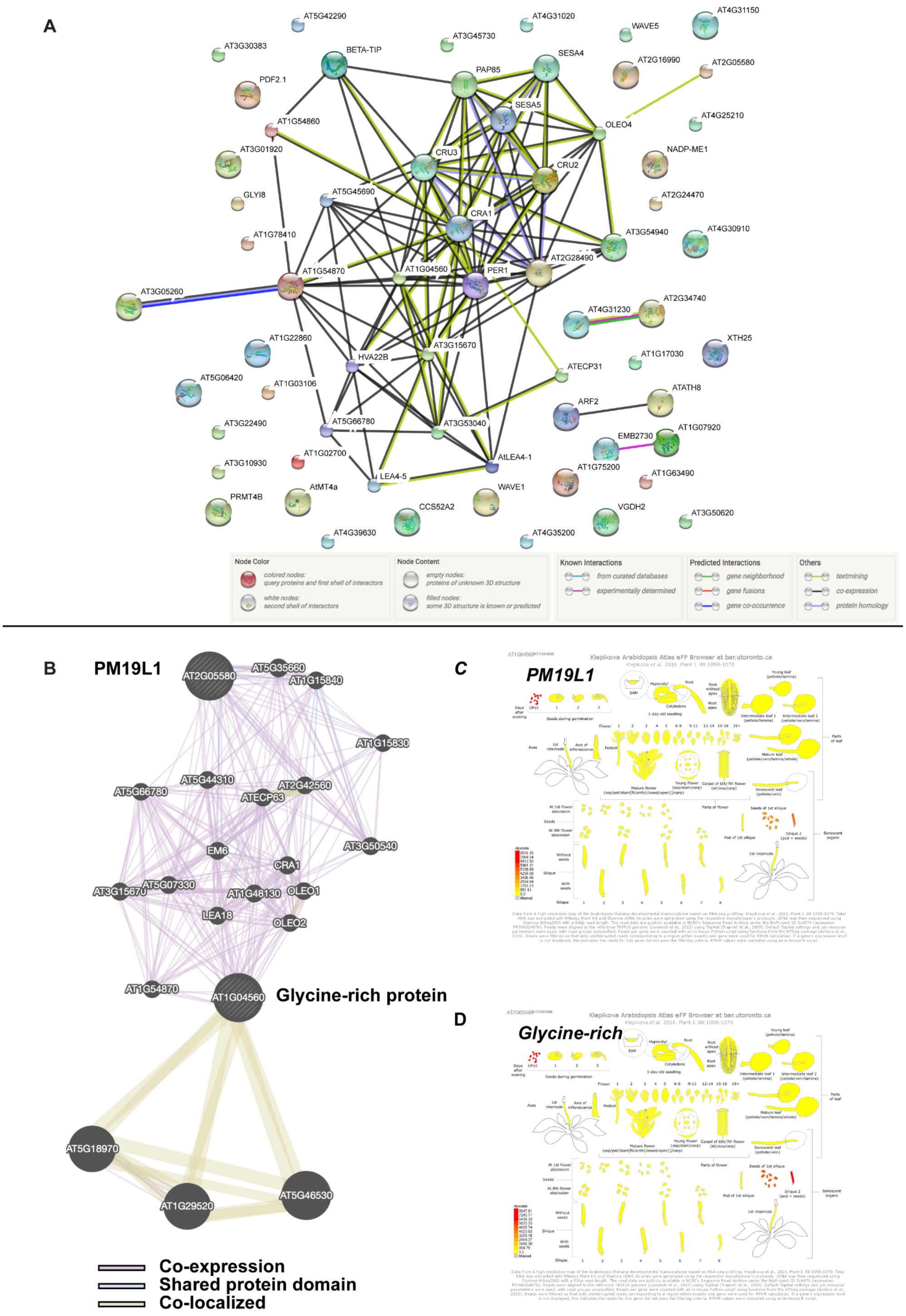

Supplementary Figure S9. Network database comparisons for genes predicted from SeedTransNet.

A. String server is used to infer gene linkage for the nodes identified in Module 3. Node color, content and interaction type represented by different edges colors are shown.

B. GeneMANIA database is used to infer the association of PM19L1 and Glycine-Rich Protein genes identified from SeedTransNet in current study.

C. Development dependent gene expression profile of PM19L1

D. Glycine-Rich Protein in eFP browser at Bar server.

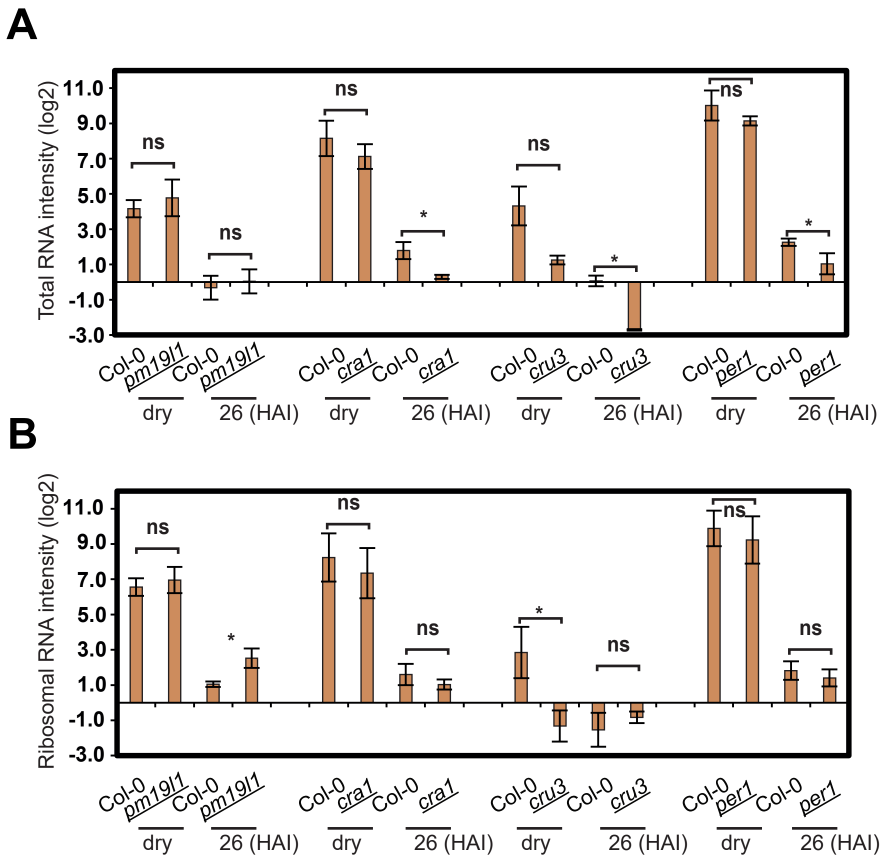

Supplementary Figure S10. Ribosomal occupancy changes of target genes determined by SeedTransNet.
The combined data is presented in figure 5D. Target genes and putative regulators are predicted from SeedTransNet. RNA levels are determined by qPCR analysis.
A) Total RNA levels of target genes in predicted regulator mutants.
B) Levels of mRNAs of target genes associated to ribosomes in predicted regulator mutants.

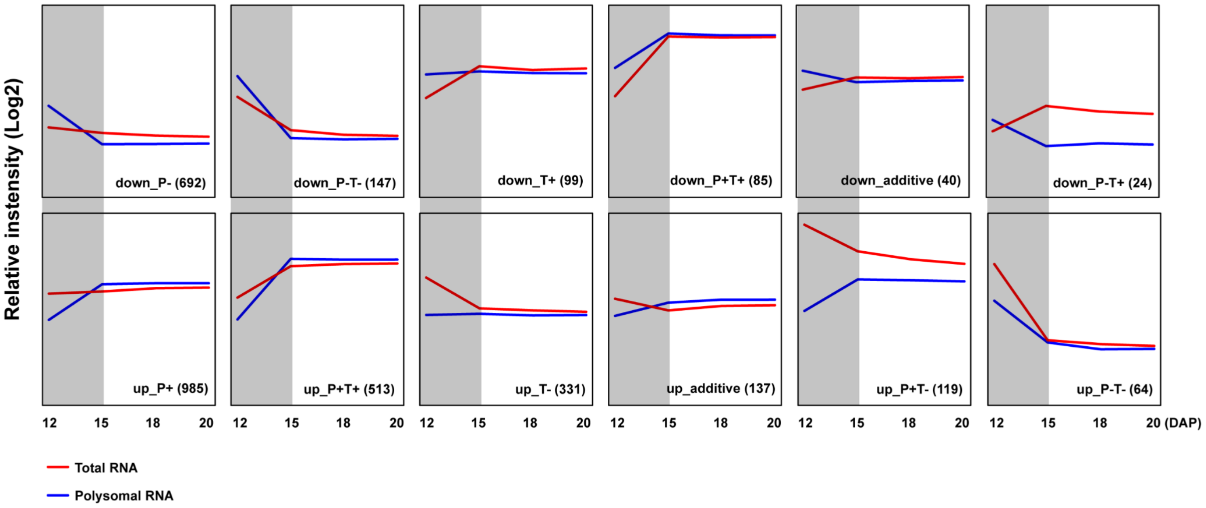

Supplementary Figure S11. Intensity profiles of genes with changed PO.

Maturation Translational Shift genes are divided into subgroups based on their significant transcriptional and translational changes from 12 to 15 DAF. In total, twelve subgroups are identified with their corresponding expression profiles following seed maturation and genes numbers under each subgroup are listed in bracket. T+ represents that the transcription is increased while the translation is unchanged under specific shift. P+ indicates translation is increased while the total mRNA abundance is unchanged. T+/P+ indicates both transcription and translation are enhanced and additive effect (Additive) means that the change of gene expression in either transcription or translation level is not significant while the additive effect of two levels contributes to the significant change of PO. The opposite effect is represented by T-, P- and T-/P- in the corresponding subgroups.

Supplementary Table S1. Primers and vectors used in this study

Primer name	primer sequence	Function
1Cxmotif	aacaGGTCTCa ggct caaca AACAAAAAAA tcagaGAGACCtgtt	motif synthesis
1Cxmotif_control	aacaGGTCTCa ggct caaca TAGCTATGCA tcagaGAGACCtgtt	Control motif synthesis
D_LuciferaseF_	aacaGGTCTCa tcag gt ATGGAAGACGCCAAAAAC	Luciferase cloning
D_LuciferaseR	aacaGGTCTCt ctga TTACACGGCGATCTTTCC	Luciferase cloning
NbUbi3_F_PCR	GCCGACTACAACATCCAGAAGG	ubiquitin control qPCR
NbUbi3_R_PCR	TGCAACACAGCGTTAACC	ubiquitin control qPCR
LuciferaseF_qPCR	AGCTCCCAATCATCCAAAAA	Luciferase qPCR
LuciferaseR_qPCR	AAGGACTCTGGCACAAAA	Luciferase qPCR
Clycinrich_F_qPCR	CATCAAGGTTGTTGCCTCCATCG	Glycine rich protein qPCR
Clycinrich_R_qPCR	GCATGTCGACTTTGACTCCTCCAC	Glycine rich protein qPCR
AWPMqPCR_F	GGAATGATAGTTTAGCCGCTG	pm19L1 qPCR
AWPMqPCR_R	CCTCTCCATCCTCCTATGTTT	pm19L1 qPCR

Vector name	vector function
PGGA004	Greengate vetor A cassette with 35S pro
PGGB003	Greengate vetor B cassette with dummy seq (resulting in empty site)
PGGC000	Greengate vetor C cassette empty
PGGD000	Greengate vetor D cassette empty
PGGE001	Greengate strain E cassette with RbCS terminator
PGGF007	Greengate vetor F cassette with Kan+R
PGGZ001	Greengate strain desitination vector

Supplementary Table S2. Transgenes and mutants used in this study

Mutant name	Salk ID	TAIR ID
oleosin	SALK_106678C1	AT2G25890
pm19l1	SALK_075435.31.95.x	AT1G04560
per-1	SALK_133714.51.45.x	AT1G48130
cra1	SALK_002668	AT5G44120
cru2	SALK_045987	AT1G03880
cru3	GABI-KAT 283D09	AT4G28520
lea1	SALK_017464.55.00.x	AT1G32560
lea5-1	SALK_041260.54.40.x	AT2G40170
lea5-2	SALK_112719.23.15.x	AT2G40170
dhn	SALK_042570.20.65.x	AT2G21490
swp	SALK_038352.41.15.x	AT3G22490

Supplementary Data Sets (available in separate excel file)

Supplementary Data Set 1. Raw normalized intensity levels for total RNA (T) and polysomal RNA (P) (log_2 transformed) during seed maturation.

Supplementary Data Set 2. Genes translationally regulated during seed maturation.

Supplementary Data Set 3. GO enrichment analysis of genes translationally regulated during seed maturation.

Supplementary Data Set 4. GO terms enriched among the 100 genes with the highest structure scores (within the CDS) of the genes with PO during the seed maturation shift.

Supplementary Data Set 5. Raw normalized intensity levels for total RNA (T) and polysomal RNA (P) (log2 transformed) for combined dataset (maturation and germination).

Supplementary Data Set 6. Transcript clusters based on the dynamics in polysome occupancy during seed maturation and germination.

Supplementary Data Set 7. GO enrichment analysis of the genes in the individual clusters from hierarchical clustering of PO time course during seed maturation and germination (Figure 3).

Supplementary Data Set 8. Seed translation network (SeedTransNet). Columns listed are resource, target, linkage weight, direction for the network.

Supplementary Data Set 9. Infomap community prediction and gene linkage inference by SeedTransNet.

Supplementary Data Set 10. GO enrichment analysis of genes in SeedTransNet modules

Supplementary Data Set 11. Genes included in the LEA protein network as identified in Module 3 of SeedTransNet.

Supplementary Data Set 12. Genes included in the ABI3 core regulon network as identified in Module 3 of SeedTransNet.

Supplementary Data Set 13. Raw normalized signals in maturation dataset of the known seed maturation related genes.

Supplementary Data Set 14. Raw data used for figures.

References

Angelovici, R., Fait, A., Zhu, X., Szymanski, J., Feldmesser, E., Fernie, A.R., and Galili, G. (2009). Deciphering transcriptional and metabolic networks associated with lysine metabolism during Arabidopsis seed development. Plant Physiol **151,** 2058-2072.

Branco-Price, C., Kawaguchi, R., Ferreira, R.B., and Bailey-Serres, J. (2005). Genome-wide analysis of transcript abundance and translation in Arabidopsis seedlings subjected to oxygen deprivation. Ann Bot **96,** 647-660.

Juntawong, P., and Bailey-Serres, J. (2012). Dynamic Light Regulation of Translation Status in Arabidopsis thaliana. Frontiers in plant science **3,** 66.

Li, F., Zheng, Q., Vandivier, L.E., Willmann, M.R., Chen, Y., and Gregory, B.D. (2012). Regulatory impact of RNA secondary structure across the Arabidopsis transcriptome. Plant Cell **24,** 4346-4359.

Monke, G., Seifert, M., Keilwagen, J., Mohr, M., Grosse, I., Hahnel, U., Junker, A., Weisshaar, B., Conrad, U., Baumlein, H., and Altschmied, L. (2012). Toward the identification and regulation of the Arabidopsis thaliana ABI3 regulon. Nucleic Acids Res **40,** 8240-8254.

Righetti, K., Vu, J.L., Pelletier, S., Vu, B.L., Glaab, E., Lalanne, D., Pasha, A., Patel, R.V., Provart, N.J., Verdier, J., Leprince, O., and Buitink, J. (2015). Inference of Longevity-Related Genes from a Robust Coexpression Network of Seed Maturation Identifies Regulators Linking Seed Storability to Biotic Defense-Related Pathways. Plant Cell **27,** 2692-2708.

Sorenson, R., and Bailey-Serres, J. (2014). Selective mRNA sequestration by OLIGOURIDYLATE-BINDING PROTEIN 1 contributes to translational control during hypoxia in Arabidopsis. Proc Natl Acad Sci U S A **111,** 2373-2378.
